# Who's on base? Revealing the catalytic mechanism of inverting family 6 glycoside hydrolases†
†Electronic supplementary information (ESI) available: Detailed computational procedures including the list of collective variables screened by likelihood maximization; additional results from *Tr*Cel6A D175A mutant hydrolysis simulations; movies of hydrolysis for the *Tr*Cel6A WT and D175A mutant; “pre-slide” and “slide” conformation snapshots from the US simulations for processivity overlaid on their reference crystal structures; additional results for glucose–protein interactions during processivity; and a movie of substrate processivity. See DOI: 10.1039/c6sc00571c


**DOI:** 10.1039/c6sc00571c

**Published:** 2016-06-01

**Authors:** Heather B. Mayes, Brandon C. Knott, Michael F. Crowley, Linda J. Broadbelt, Jerry Ståhlberg, Gregg T. Beckham

**Affiliations:** a Department of Chemical and Biological Engineering , Northwestern University , Evanston , IL 60208 , USA; b National Bioenergy Center , National Renewable Energy Laboratory , Golden , CO 80401 , USA . Email: gregg.beckham@nrel.gov; c Biosciences Center , National Renewable Energy Laboratory , Golden , CO 80401 , USA; d Department of Chemistry and Biotechnology , Swedish University of Agricultural Sciences , SE-75007 , Uppsala , Sweden . Email: jerry.stahlberg@slu.se

## Abstract

In several important classes of inverting carbohydrate-active enzymes, the identity of the catalytic base remains elusive, including in family 6 Glycoside Hydrolase (GH6) enzymes, which are key components of cellulase cocktails for cellulose depolymerization. Despite many structural and kinetic studies with both wild-type and mutant enzymes, especially on the *Trichoderma reesei* (*Hypocrea jecorina*) GH6 cellulase (*Tr*Cel6A), the catalytic base in the single displacement inverting mechanism has not been definitively identified in the GH6 family. Here, we employ transition path sampling to gain insight into the catalytic mechanism, which provides unbiased atomic-level understanding of key order parameters involved in cleaving the strong glycosidic bond. Our hybrid quantum mechanics and molecular mechanics (QM/MM) simulations reveal a network of hydrogen bonding that aligns two active site water molecules that play key roles in hydrolysis: one water molecule drives the reaction by nucleophilic attack on the substrate and a second shuttles a proton to the putative base (D175) *via* a short water wire. We also investigated the case where the putative base is mutated to an alanine, an enzyme that is experimentally still partially active. The simulations predict that proton hopping along a water wire *via* a Grotthuss mechanism provides a mechanism of catalytic rescue. Further simulations reveal that substrate processive motion is ‘driven’ by strong electrostatic interactions with the protein at the product sites and that the –1 sugar adopts a ^2^S_O_ ring configuration as it reaches its binding site. This work thus elucidates previously elusive steps in the processive catalytic mechanism of this important class of enzymes.

## Abstract 2

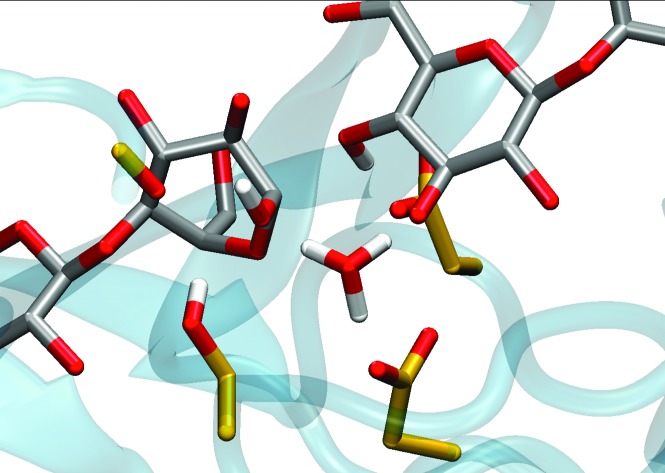
Unbiased simulations reveal a water wire enabling and rescuing the catalytic base of an inverting glycoside hydrolase.

## Introduction

Glycoside hydrolase (GH) enzymes are produced by all kingdoms of life.^1^,^2^ They play vital roles in the turnover of carbon in nature,^3^ fundamental mechanisms of cell biology, industrial biotechnology, and human health.^4^–^7^ These ubiquitous enzymes are currently categorized into 135 families, catalogued on the Carbohydrate Active Enzymes database (CAZy, ; http://www.cazy.org).^1^,^2^ One particularly important role of GH enzymes involves the turnover of carbohydrates, such as cellulose, hemicellulose, and chitin in the biosphere, where enzymes from multiple GH families are secreted by microbes to produce sugars for food. Industrially, these natural systems are often starting points for designer enzyme cocktails to conduct cellulose depolymerization, with the ultimate aim of producing sugars for the production of renewable chemicals and fuels.^8^ Given their fundamental and industrial importance, studying the elementary mechanisms of cellulose depolymerization could suggest methods for improving efficiency, which may profoundly impact the availability and affordability of cellulosic biofuels.^3^,^9^ While significant advances have been made in characterizing cellulolytic GHs, many questions regarding their structure and function remain unanswered.^3^

Based on experimental observations of stereochemistry, Koshland postulated two general hydrolytic mechanisms that GHs employ for enzymatic action on anomeric carbon atoms: a single-displacement inverting mechanism or a double-displacement retaining mechanism.^10^ In the first step of the double-displacement mechanism, a nucleophilic enzyme residue attacks the anomeric carbon while an acidic residue donates a proton to the glycosidic oxygen. In the second step, a water molecule attacks the anomeric carbon such that its hydroxide group bonds to the anomeric carbon, restoring the original stereochemistry and causing deglycosylation, while its proton transfers to the acidic residue, resetting the active site charge distribution for subsequent catalysis. Conversely, in the single-displacement mechanism, the water molecule attacks the anomeric carbon simultaneously with the acidic residue, donating a proton to the glycosidic oxygen and inverting the stereochemistry at the anomeric carbon. The required positioning of the attacking water and acidic residues prevents the proton of the attacking water from resetting the charge of the acidic residue in this single step. An acceptor must absorb the proton, and the active site charge distribution must be reset in a separate step.

Almost all known GH enzymes follow one of these two proposed mechanisms involving protein residues serving as catalytic acids and bases,^3^,^11^ and identifying which parts of the enzymes serve as proton acceptors and proton donors is an active area of investigation.^12^–^14^ Simulation can complement these experimental advances by providing dynamic, spatiotemporal models of enzyme mechanisms.^15^–^17^ These models can test hypotheses and provide atomic-level insights toward the development of structure–function relationships.

Polysaccharide-active GHs are also characterized according to whether they perform non-processive chain hydrolysis to create new chain ends or processively cleave glycosidic bonds along a polymer chain.^3^ These activities are synergistic, with the bulk of hydrolysis accomplished *via* processive action due to the efficiency of hydrolyzing multiple bonds in between enzyme-substrate binding and unbinding.^18^,^19^ Recently, our group identified the molecular details of the catalytic^20^ and processive^21^ action of the retaining GH *T. reesei* Cel7A (*Tr*Cel7A), the main component of industrial enzyme cocktails for cellulose decomposition. The catalytic mechanism study identified key, non-intuitive contributions to the reaction coordinate (RC) including protein side-chain conformational changes and a product-assisted step. The processivity study revealed that electrostatic interactions with the leading substrate glycosyl ring provide the driving force for chain translocation.

**Fig. 1:**
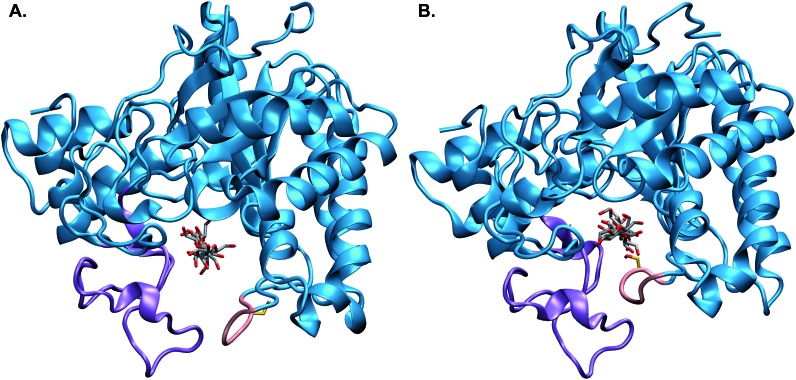
The catalytic domain of *Tr*Cel6A showing the substrate binding-site tunnel enclosed by the N-terminal loop (purple) and the active-center (C-terminal) loop (pink) in the (A) “open” and (B) “closed” positions, with the entrance to the active site tunnel in the foreground. These images come from the processivity and hydrolysis simulations, respectively, using models based on crystal structures as described in the Computational methods.

**Fig. 2:**
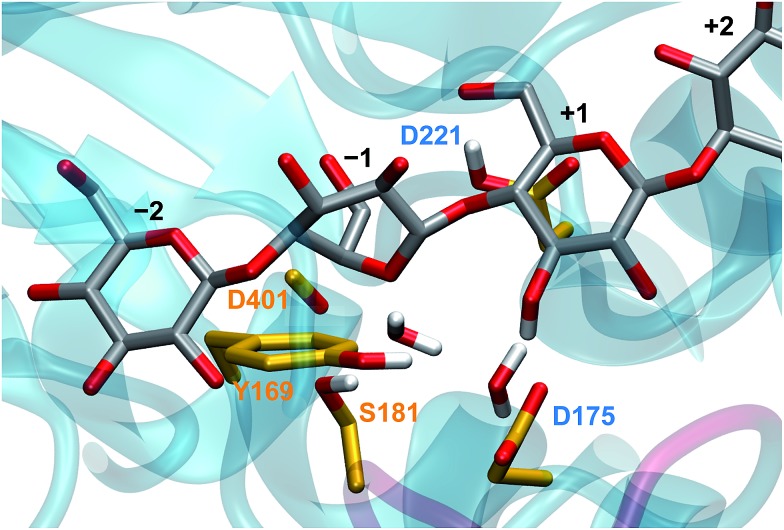
The *Tr*Cel6A active site from the hydrolysis simulations, based on crystal structures as described in the Computational methods, several key residues and substrate binding sites labeled according to the conventional scheme.^41^,^42^ The two water molecules shown are present in the crystal structure.

Additional computational studies validated Koshland's postulated mechanisms for other GH^22^,^23^ and glycosyltransferase (GT) enzymes.^24^–^26^ Yet, detailed mechanisms of other key GH enzymes have remained elusive, including the inverting *Tr*Cel6A (formerly designated CBHII^27^) shown in Fig. 1.^3^,^28^,^29^ This enzyme is industrially significant due to its synergistic action with *Tr*Cel7A.^8^,^30^–^32^ *Tr*Cel6A was the first reported fungal cellulase crystal structure,^33^ and since that time, GH6 cellulases have been the subject of multiple structure–function studies to elucidate the catalytic mechanism.^3^ These studies led to the hypothesis of a four-step processive catalytic cycle involving (1) procession of the substrate into the active site, which occurs with the active site loops in the “open” position (Fig. 1A), (2) loop closure/activation, (3) hydrolysis with the active site loops in the “closed” position (Fig. 1B), and (4) product expulsion/loop opening.^3^,^29^

Experimental studies confirmed that D221 (shown in Fig. 2) acts as the catalytic acid,^34^ yet the identification of its catalytic base has remained elusive, even leading to speculation that *Tr*Cel6A does not employ a proton-accepting residue (catalytic base).^35^ Koivula *et al.*^34^ proposed that the D175 residue can accept the excess proton through a short water wire (one bridge water molecule between the nucleophilic water and D175), rather than directly from the nucleophilic water, *via* the Grotthuss mechanism.^36^ Water molecules have been resolves in the active-site of the substrate-bound *Tr*Cel6A^37^ and *Humicola insolens* Cel6A (*Hi*Cel6A),^38^ in accordance with this hypothesis. Studies of *Thermobifida fusca* Cel6B (*Tf*Cel6B) are also consistent with involvement of a proton-shuttling network,^39^ including identification of two water molecules in the crystal structure of substrate-bound WT enzyme, leading Sandgren *et al.* to propose that a Grotthuss mechanism is also responsible for proton transfer in this bacterial member of the GH6 family.^40^ However, Koivula *et al.*^34^ also demonstrated that the enzyme retained 2–3% residual activity when the aspartic acid is mutated to the non-proton accepting alanine, rather than the null activity expected if D175 acted as a traditional GH catalytic base. A proposed mechanism to explain the residual activity is catalytic rescue by a longer water wire shuttling the excess proton to another proton acceptor or even to bulk water.^3^,^29^

To investigate this intriguing mechanism, we used a QM/MM molecular dynamics (MD) to simulate the catalytic domain of *Tr*Cel6A, allowing us to model bond rearrangement at the catalytic center of the enzyme. To elucidate the RC, we employed aimless shooting (AS) with likelihood maximization.^43^,^44^ This transition path sampling (TPS) method^45^ explores the transition state (TS) ensemble without modifying the simulation by bias potential along a preconceived RC.^46^ The potential of mean force (PMF), which describes how the free energy changes along the RC, for the reaction was then determined by equilibrium path sampling (EPS).^47^ We also performed simulations of the *Tr*Cel6A D175A mutant to investigate mechanisms of catalytic rescue to account for the observed residual activity after mutation of the putative base.

**Fig. 3:**
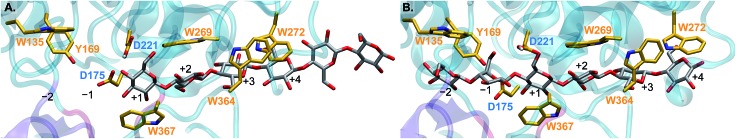
Cross-sectional view of the *Tr*Cel6A tunnel in the (A) “pre-slide” and (B) “slide” modes from processivity simulations performed in this work as described below. Aromatic residues in the tunnel that interact with the substrate are shown and labeled in orange, and the substrate binding sites are labeled in black. The residues proposed to donate (D221) and accept (D175) a proton in the hydrolysis reaction are labeled in blue. Hydrolysis cleaves the glycosidic bond joining the glycosyl units at the –1 and +1 substrate binding sites.

Furthermore, we simulated the processive action wherein, with the active site loops in the “open” position (Fig. 1A), the substrate advances from its position immediately after hydrolysis and product expulsion (the “pre-slide” mode, Fig. 3A) into position for the next catalytic event (the “slide” mode, Fig. 3B).^3^,^29^ Carbohydrate–aromatic interactions in the tunnel have been reported to play key roles in processivity,^18^,^29^,^48^–^51^ and are shown and labeled in orange. The aspartic acid residues 175 and 221 are labeled in blue to indicate where the hydrolysis reaction will take place. The black labels identify the substrate binding sites +1 through +4.^3^ The substrate enters from the right side of the figure (binding site +4). Binding sites –2 and –1 are occupied in the “slide” mode but not in the “pre-slide” mode. Hydrolysis breaks the glycosidic bond between the –1 and +1 binding sites.

These simulations allow us to calculate energy barriers and identify the interactions for these two important parts of the catalytic cycle. In combination with previous studies of product expulsion^52^ and active site loop opening and closing,^37^,^53^ this work greatly expands our understanding of the catalytic cycle of this industrially important enzyme.^3^,^29^ Additionally, the findings presented here are expected to aid advances in the understanding of cellulase function and to apply to other inverting carbohydrate-active enzymes. As previously noted, while the mechanisms for some inverting GH enzymes have been reported,^22^,^23^ they have been elusive for others beyond those in GH6, such as for the *Clostridium thermocellum* Cel124 inverting cellulase *endo*-β-1,4-glucanase, and it has been suggested that the water wire hypothesized in GH6 hydrolysis could also enable hydrolysis in the GH124 family enzymes.^54^ Furthermore, identifying catalytic residues for some inverting glycosyltransferases^55^–^57^ and DNA glycosylases^58^ have also proven elusive, sparking debate and prompting analogies with *Tr*Cel6A.^58^,^59^

## Computational methods

Detailed procedures are documented in the ESI† with an overview provided here. The CHARMM^60^ package was used to build and equilibrate all systems and for some trajectory analyses. The CHARMM36 force field was used for all simulations and all parts of the system: the enzyme,^61^–^63^ carbohydrate ligands,^64^,^65^ and sodium ions^66^ with the TIP3P model for water.^67^ All simulations were conducted at 300 K. After initial minimization and heating steps in the NVT ensemble, the remaining simulations employed the NPT ensemble with the pressure set to 1.0 bar.

For the hydrolysis simulations, the *Tr*Cel6A Michaelis complex was based on Protein Data Bank (PDB)^68^ ID ; 1QJW 37 (*Tr*Cel6A Y169F in complex with Glc_2_–S–Glc_2_; with closed active-center loop) with the following modifications: the addition of the Tyr85 residue from PDB ID ; 1QK2 37 (*Tr*Cel6A WT in complex with Glc_2_–S–Glc_2_; with open active-center loop); replacing the mutant Phe169 with the Tyr169 from PDB ID ; 1QK2; using the Asp221 conformation from PDB ID ; 1HGW 34 (apo *Tr*Cel6A D175A mutant; with open active-center loop); and using the cellohexaose ligand from PDB ID ; 4AVO 69 (*Tf*Cel6B) D274A; with open active-center loop), with the leading four glycosyl units aligned to the tetramer in PDB ID ; 1QJW. This model was used to generate the mutant D175A by replacing Asp175 with Ala175 from crystal structure ; 1HGW of *Tr*Cel6A D175A.^34^ For the processivity study, the *Tr*Cel6A “slide” and “pre-slide” conformations were based on PDB ID ; 1QK2 and the cellohexaose ligand from PDB ID ; 4AVO. For the “pre-slide” mode, the –1 and –2 ligand monomers were removed, and monomers were added in the +5 and +6 positions, in the same starting geometry as the +3 and +4 monomers. After the initial equilibration in CHARMM, the remaining simulations were primarily performed using the Amber^70^ software package version 12. Some unrestrained MM-only simulations were run in NAMD^71^ starting from the minimized and equilibrated structures from CHARMM.

In the hydrolysis simulations, the QM region was modeled with the self-consistent charge density functional tight-binding (SCC-DFTB) method with second-order terms in the charge density fluctuations.^72^,^73^ The choice of this method is discussed in the ESI.† The ESI† also details the procedures employed for AS, likelihood maximization, committor analysis, and EPS. The processivity simulations employed umbrella sampling (US)^74^ and the weighted histogram analysis method (WHAM)^75^–^77^ to produce the PMF, with further details included in the ESI.† Images of the proteins were created in VMD.^78^ Additional tools utilized in this work include CPPTRAJ^79^ and CHAMBER13.^80^ Plots were created in Igor Pro version 6.36 (Wavemetrics, Lake Oswego, OR, USA), with data for electrostatic and van der Waals interactions smoothed using the binomial algorithm with 250 passes over the 12 535 data points created by averaging the energy values within the collective variable (CV) bins of width 0.001 Å.

## Results and discussion

### WT hydrolysis

**Fig. 4:**
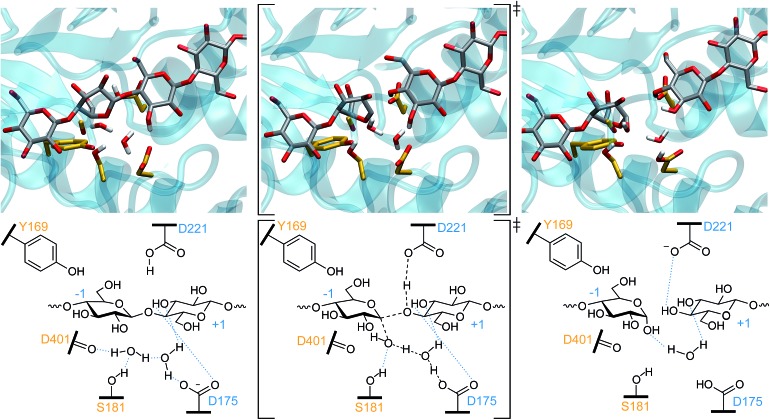
The one-step inverting hydrolysis reaction by *Tr*Cel6A. In the reactant conformation (left), the –1 glucopyranoside ring is in a ^2^S_O_ conformation. It is in a ^2,5^B conformation in both the TS (middle) and product state (right). Intermediate bond lengths are indicated in the TS by dashed black lines. Blue dotted lines indicate hydrogen bonding.

For *Tr*Cel6A to follow the canonical inverting mechanism, eight bond rearrangements (four cleaved and four formed, see Fig. 4) must occur in one step. As described in detail in the ESI,† simulation of this reaction began with biased simulations to create 48 potential TS structures, based on a range of interatomic distances for the eight bond rearrangements involving the WT protein, the substrate, and two active site water molecules. 37 of these hypothesized structures yielded “reactive” trajectories linking the reaction and product structures shown in Fig. 4, supporting the hypothesis that D175 can accept the proton from the nucleophilic water *via* a second bridge water (Fig. 4). The biased MD simulations were followed with unbiased AS simulations allowing collection of thousands of conformations of the TS ensemble. As noted in the Computational methods, these TPS simulations use a Hamiltonian that is not biased along a preconceived RC, as employed by methods such as US^74^ or metadynamics.^81^

We used likelihood maximization to test 87 order parameters (OPs), such as atomic distances or dihedral angles, to determine which OP or combination of OPs best represent the RC (see the ESI† for the full list of OPs tested). We excluded data from the first 500 AS points of each run to allow for decorrelation from the initial TS guesses. Another 2000 points were obtained in each of 32 independent runs. Likelihood maximization was used to identify the best RC and determine its parameters using half or all of the 64 000 resulting points to test for converged AS simulations and RC identification. A list of the best scoring one-, two-, and three-parameter RCs is included in the ESI.†

**Fig. 5:**
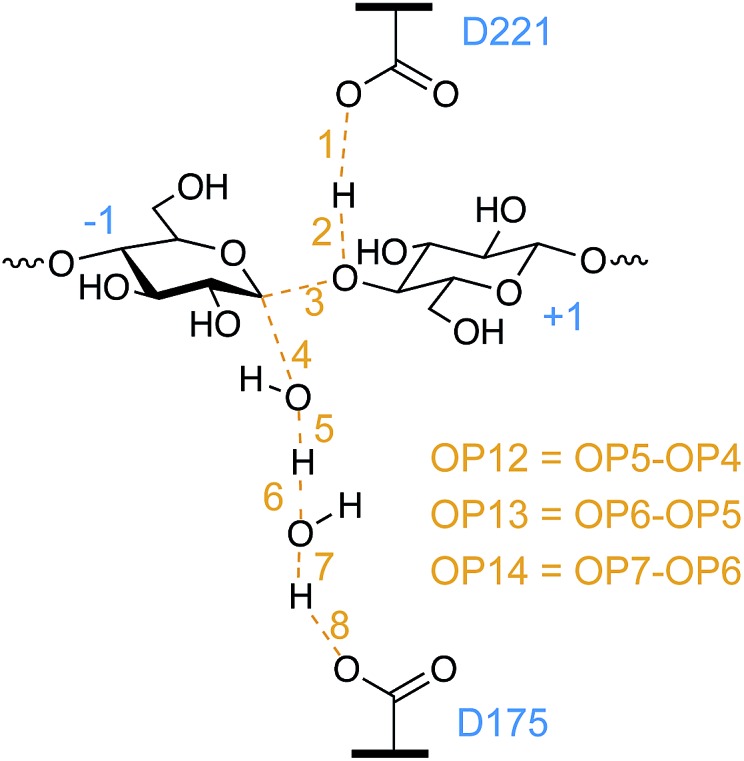
Of the 87 OPs tested and listed in the ESI,† only OPs 4, 5, 6, 12, and 13 were identified by likelihood maximization as components of the best one-, two-, or three-parameter RCs.

Of the set of tested OPs, the only ones for which inclusion in the RC significantly improved the likelihood score (as measured by the Bayesian information criterion^43^,^82^) were distances between atoms with bonds forming and breaking which included at least one atom of the nucleophilic water. The key distances identified are captured in the best identified two-parameter RC, OP6 and OP12 (Fig. 5). As shown, OP12 is a difference of distances involving the nucleophilic water oxygen: the distance between the oxygen atom and one hydrogen nucleus of the nucleophilic water (bond breaking), minus the distance between the nucleophilic water oxygen and the –1 glucopyranoside anomeric carbon (bond forming). OP6 is the distance between the hydrogen nucleus and the bridge water oxygen atom (bond forming). Committor analysis^83^ was performed with this RC (a linear combination of OP6 and OP12), resulting in a relatively flat histogram. Sampling errors and the complexity of the RC likely contribute to deviations from an ideal committor.^20^,^84^ To correct for the resulting overestimation of the rate coefficient,^85^ we calculated the transmission coefficient for this RC, as discussed below.

While it was not surprising that the key distances involve the nucleophilic oxygen, it is surprising that the likelihood of the RC did not improve with the inclusion of OPs describing hydrogen bonding between water molecules and the D401 carbonyl or S181 hydroxyl group. Our simulations show that hydrogen bonding between the nucleophilic water and the S181 side chain and D401 backbone position the nucleophilic water for attack in the reactant conformation, consistent with the postulated roles for these residues.^3^ As the distance between the nucleophilic water oxygen and the anomeric carbon decreases and the system moves to the TS conformation, the nucleophilic water oxygen is no longer close enough to the D401 carbonyl oxygen to allow for hydrogen bonding, but it remains hydrogen-bonded to the S181 hydroxyl group. The reactant conformation of the bridge water molecule is aligned by hydrogen bonding with a D175 carboxyl hydrogen, the bridge water, and with OH3 on the +1 glycosyl unit. While this hydrogen bonding appears to be crucial for positioning the water molecules for attack, the putative RC indicates that the nucleophilic water oxygen bond-forming and bond-cleaving distances better describe the hydrolysis reaction.

**Fig. 6:**
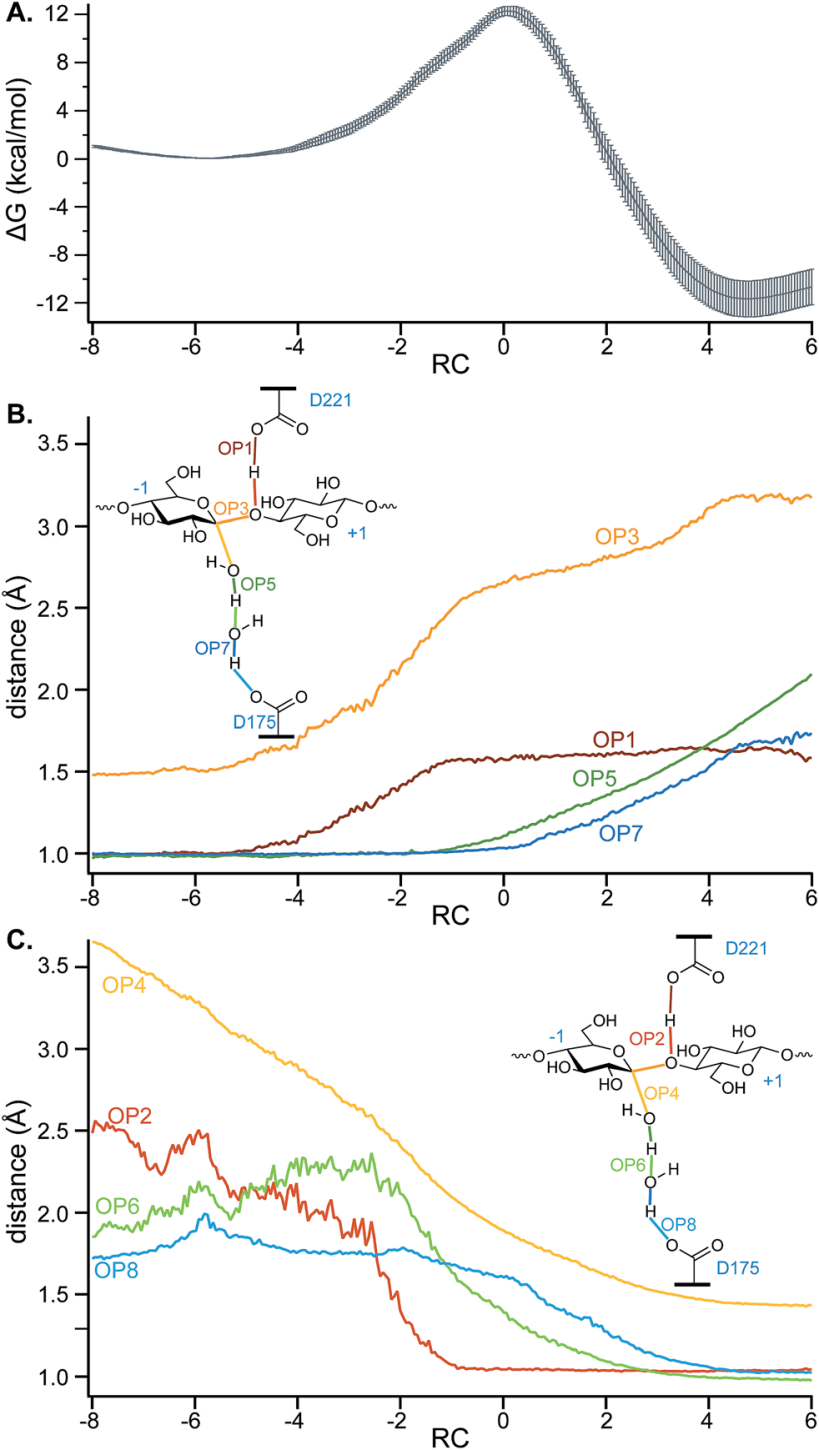
(A) The average PMF (after setting the reactant energy to zero) and standard error generated from five independent EPS simulations using the two-parameter RC (unitless; a linear combination of OP6 and OP12, which is the difference between OP5 and OP4). A subset of the conformations generated in the EPS simulations were used to calculate, for the range of RC values aligning with the PMF, average values of OPs corresponding to distances between atoms of (B) breaking bonds and of (C) forming bonds.

Fig. 6 shows the PMF and the values of key OPs (distances between atoms which have bonds that form or break) along the putative RC calculated using the data from five EPS runs. As discussed above, the RC is a linear combination of the OP6 and OP12, which describe nucleophilic attack. Note that the RC is dimensionless. The zero value of the RC aligns to the TS. In this case, the reactant basin corresponds to an RC value of –5.8 and the product basin to a value of 4.7. The scale is a function of a range of OP values for the coordinates used in likelihood maximization and has no inherent significance. The larger absolute value of the RC at the reactant well relative to the product well corresponds to a late TS.

Fig. 6A shows a free energy barrier of 12.3 ± 0.4 kcal mol^–1^ is consistent with that found for the hydrolysis (deglycosylation) of *Tr*Cel7A,^20^ which was found to be 11.6 kcal mol^–1^ using the same Hamiltonian (CHARMM36 with SCC-DFTB for the QM region). The Δ*G*_rxn_ for *Tr*Cel6A is –12 ± 1 kcal mol^–1^.

Based on simulations using the FF99SB Amber32 and GLYCAM06 force fields with PBE functional, Petersen *et al.*^22^ estimated a free energy barrier of 36 kcal mol^–1^ and Δ*G*_rxn_ of approximately –12 kcal mol^–1^ for the *C. thermocellum* inverting GH8 *endo*-1,4-glucanase A, following the classic inverting mechanism in which the proton from the attacking, nucleophilic water directly transfers to the catalytic base, rather than through a bridge water as shown here for *Tr*Cel6A. While some difference in the energy barriers is expected due to the different Hamiltonians used and differences between the enzymes, their reported barrier is likely a significant overestimate; transition state theory, assuming no recrossing and thus a transmission coefficient of 1.0, yields an upper-bound rate coefficient of 3.6 × 10^–14^ s^–1^ at 300 K for a Δ*G*^‡^ of 36 kcal mol^–1^, for a timescale much longer than the experimentally observed rate. For a Δ*G*^‡^ of 12.3 kcal mol^–1^, transition state theory predicts an upper-bound rate coefficient 17 orders of magnitude larger than for a Δ*G*^‡^ of 36 kcal mol^–1^, with a timescale on the order of 100 ms. Our calculated rate coefficient including recrossing is reported below, along with the results of evaluating our TS ensemble by the histogram test.

To gain further insights into the structure of the TS, the values of OPs 1–8 (Fig. 5) were calculated for a subset of conformations generated during the EPS simulations (from 18 000 configurations) to allow calculation of the average values of these OPs in each of the bins of RC values created to calculate the PMF. For greater clarity, the OP values as a function of the RC are shown in two panels: Fig. 6B shows those that correspond to bond breaking and Fig. 6C for bond formation. The PMF indicates an overall single-barrier reaction, yet the eight bond cleavage and formation events do not occur simultaneously, as indicated both by the projection of distances onto the RC (Fig. 6B and C) and by examining the simulations (a movie for one reactive trajectory is included in the ESI†). Instead, bond rearrangement occurs in a coordinated, step-wise fashion. The leading events are the elongation of the D221 carboxylic oxygen to acidic hydrogen bond, the shortening of the distance between the acidic hydrogen and glycosidic oxygen, the elongation of the glycosidic bond, and the approach of the nucleophilic water oxygen toward the –1 glucopyranoside anomeric carbon. The beginnings of the changes align with the beginnings of the rise in free energy along the RC from the reactant well located at RC = –5.8. Table 1 reports the progress of the key order parameters OP1–8 at the peak energy on the PMF, coincident with RC = 0. The D221 carboxylic oxygen to acidic hydrogen bond (OP1) is 91% along a change in distance from 1.00 to 1.63 Å, with the distance between the acidic hydrogen and accepting glycosidic oxygen (OP2) essentially already at its final distance and the glycosidic bond mostly broken (OP3), indicating a late TS. In contrast, the O–H bonds of the nucleophilic and bridge waters are only slightly elongated (OP5 at 15% and OP7 at 7%). This phenomenon highlights the crucial role of transferring the protons in the water wire for the reaction to proceed. It also underscores the importance of the alignment of the two active site water molecules for nucleophilic attack and proton transfer, as they must be in position on the reaction conformation for the reaction to proceed.

**Table 1:** Values of OP1–8 with their standard deviations in Å, at the reactant basin, putative TS, and product basin, and the percent change in distance from the reactant to TS compared to the change in distance from the reactant to product. For reference, the values of the RC and Δ*G* in kcal mol^–1^ (compared to the reactant basin and reported with their standard error) are included

	RC	Δ*G*	OP1	OP2	OP3	OP4	OP5	OP6	OP7	OP8
Reactant basin	–5.8	0.0	1.00 ± 0.03	2.48 ± 0.62	1.50 ± 0.05	3.24 ± 0.13	0.99 ± 0.03	2.16 ± 0.54	0.99 ± 0.03	1.99 ± 0.48
Putative TS	0.0	12.3 ± 0.4	1.58 ± 0.12	1.04 ± 0.04	2.66 ± 0.11	1.89 ± 0.04	1.11 ± 0.06	1.39 ± 0.12	1.04 ± 0.05	1.61 ± 0.12
Product basin	4.7	–11.7 ± 1.5	1.63 ± 0.13	1.04 ± 0.04	3.18 ± 0.19	1.45 ± 0.03	1.81 ± 0.03	0.99 ± 0.03	1.67 ± 0.15	1.03 ± 0.04
% change at TS			91%	99%	69%	75%	15%	66%	7%	40%

The low-energy product well is only reached when the transferring proton is accepted by a D175 carboxylic acid. The non-simultaneous multiple bond-cleavage and bond-formation events that depend on the participation of highly motile water molecules may all contribute to the difficulty of cleanly defining a one-dimensional RC that would yield a histogram sharply peaked at *p*_B_ = 0.5. As discussed below, we found that the simulations show that while water molecules occupy the active site as shown by the crystal structures, they are not static; the molecules can exchange positions with nearby water molecules. The unbiased simulations performed in this study confirm that D175 can indeed serve as the catalytic base. When it accepts the proton, the resulting product well is very stable, with a Δ*G*_rxn_ almost three times greater in magnitude than for *Tr*Cel7A, providing a driving force for the reaction.

The calculated transmission coefficient^86^ is 0.43. This low value likely compensates for an imperfect RC, indicated by non-ideal results of the *p*_B_ histogram test.^46^,^84^ The resulting rate coefficient of 2.9 × 10^3^ s^–1^ at 300 K calculated by transition state theory is slightly lower than the second (deglycosylation) step of the *Tr*Cel7A hydrolysis mechanism, 5.3 × 10^3^ s^–1^.^20^

**Fig. 7:**
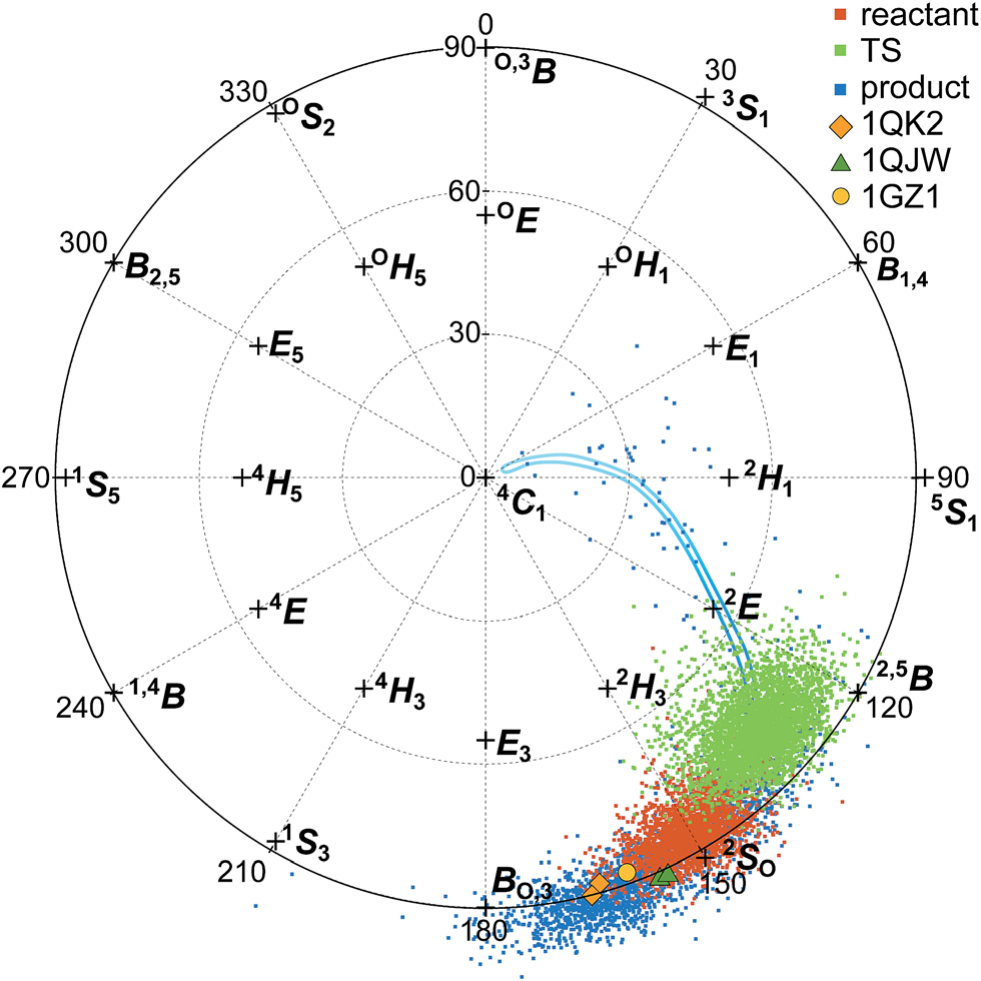
Top-down view of the northern-hemisphere of the Cremer–Pople sphere^87^ designating the –1 glucopyranoside puckering coordinates for the reactant (red), TS (green), and product (blue) for the elementary reaction shown in Fig. 4. Markers designate the puckering coordinates for the –1 glucopyranoside of the substrate mimic methyl 4-*S*-β-d-cellobiosyl-4-thio-β-d-cellobioside co-crystallized with *Tr*Cel6A wild-type (PDB ID ; 1QK2; orange diamond);^37^*Tr*Cel6A Y169F mutant (PDB ID ; 1QJW; dark green triangle);^37^ and *Hi*Cel6A D416A mutant (PDB ID ; 1GZ1; yellow circle).^88^ The two markers for PDB IDs ; 1QK2 and ; 1QJW represent the two chains present in the deposited structures. The blue line shows an approximate, smoothed ring-puckering path sampled during product ring fluctuations.

Fig. 7 shows the Cremer–Pople puckering coordinates^87^ of the –1 glucopyranoside in the reactant, TS, and product conformations for the simulated hydrolysis reaction shown in Fig. 4, harvested as described in the ESI† and displaying results from every 500th 1 fs step. Fig. 7 also shows the puckering coordinates of the glycosyl moiety in the active site –1 position for crystal structures of GH6 cellulases co-crystallized with the non-hydrolyzable substrate mimic methyl 4-*S*-β-d-cellobiosyl-4-thio-β-d-cellobioside: *Tr*Cel6A wild-type (WT) (PDB ID ; 1QK2);^37^*Tr*Cel6A Y169F mutant (PDB ID ; 1QJW);^37^ and *Hi*Cel6A D416A mutant (PDB ID ; 1GZ1).^88^

Interestingly, the puckering conformation changes along the reaction coordinate do not trace out straight path on the Cremer–Pople sphere, but a boomerang-like catalytic itinerary. The reactant structures reflected in Fig. 7 are in the Michaelis complex configuration with the –1 glucopyranoside adopting the ^2^S_O_ pucker configuration. The TS ring conformations also remain close to the Cremer–Pople sphere equator, centered between the ^2^S_O_ and the ^2,5^B configurations. The product puckering configuration then shifts west, centered between the B_O,3_ and the ^2,5^B configurations. The cleaved cellobiose product remains in the –1 binding site for the five independent, 200 ps of unbiased, unconstrained simulations performed to collect the data shown in Fig. 7. However, the –1 glucopyranoside ring shows more flexibility after cleavage, as evidenced by the wider spread of product ring conformations shown in Fig. 7. The ring readily samples the B_O,3_, ^2^S_O_, and ^2,5^B conformations, the later conformations mostly obscured in Fig. 7 by the reactant and TS conformations; this figure is reproduced as separate panels for each state in the ESI.† In one of the five simulations, the –1 glucopyranoside ring moved from the B_O,3_ conformation to the ^2^S_O_ to the ^2,5^B conformation, and then continued to the ^2^E conformation and on to ^4^C_1_, which is the most stable orientation for a glucopyranoside ring in solution.^89^ Following the trajectory for another 150 ps of QM/MM MD revealed that the ring returned to the B_O,3_/^2^S_O_/^2,5^B conformations which are primarily sampled, rather than expelling from the enzyme product site in this short time period. This smoothed, approximate path is indicated by the blue line shown in Fig. 7. The simulations of the product conformation in the active site indicate the range of conformations accessible due to normal fluctuations and provide some information about the puckering itinerary between B_O,3_ and ^4^C_1_.

The ^2^S_O_ and the ^2,5^B conformations adopted by the reactant and product –1 glycosyl moieties are among the lowest free-energy ring conformations for the β-d-glucose monomer on the equator of the Cremer–Pople sphere.^89^ The ring configurations for the –1 glycosyl moieties of the experimentally determined Michaelis structures also lie on the equator, between the canonical B_O,3_ and ^2^S_O_ coordinates. They overlap more closely with the product –1 puckering conformations than reactant conformations, although still close to the –1 puckering reactant conformation. Note that the substrates in the crystal structures contain a sulfur atom in place of the glycosidic oxygen atom connecting the –1 and +1 subunits. The larger atom may slightly perturb the position of the –1 subunit, which may in turn slightly perturb how the enzyme puckers that subunit. Still, the similarity in puckering structure demonstrates consistency between the simulations presented in this work and the experimentally reported crystal structures.

### D175A mutant hydrolysis

As previously noted, when residue 175 is no longer able to accept a proton due to its mutation into an alanine residue, 2–3% residual activity remains, prompting our investigation of whether the hydrolysis event could be simulated with the *Tr*Cel6A D175A mutant. To infer whether a proton (or proton–water complex) would be able to escape the active site, we investigated the ease with which a water molecule could escape the active site. Unrestrained MM-only simulations showed that the water molecules in the active site easily exchange positions with neighboring water molecules on a timescale of tenths of nanoseconds and diffuse into the bulk water. Protons are far more mobile than water molecules, and thus it is probable the excess proton liberated by the deprotonation of D221 could easily migrate beyond the active site, perhaps diffusing into the bulk water. Testing this hypothesis by expanding the QM region to include paths to bulk water would be prohibitively expensive. Instead, to determine whether the excess proton could hop beyond the second water in the active site, we modestly expanded the QM region to include a third water molecule initially 5.4 Å from the bridge water molecule that was also identified in the PDB ID ; 1QJW crystal structure. We excluded the mutated 175 alanine from the QM region. As noted in the detailed Computational procedures in the ESI,† we harvested sets of distances for OP1–6 from several well-equilibrated (more than 2000 trajectories completed), accepted WT AS points for use in targeted MD to create TS guesses for the D175A mutant cases. From these guesses, we were able to obtain multiple reactive trajectories for hydrolysis of the glycosidic bond.

**Fig. 8:**
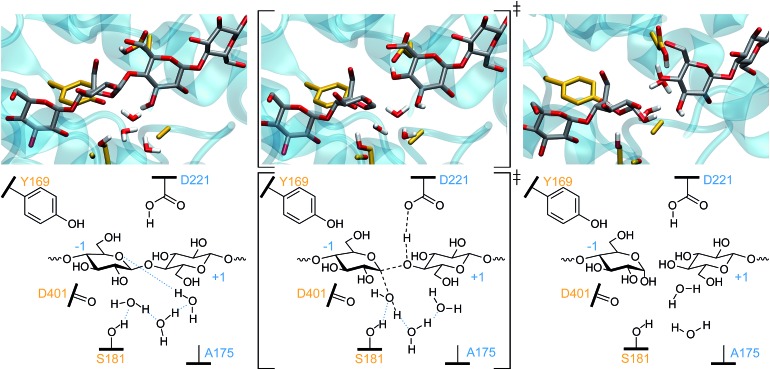
The one-step inverting hydrolysis for the *Tr*Cel6A D175A mutant. The smaller residue 175 provides additional room for the third water molecule in the reactant (left) conformation. Only the nucleophilic water has an elongated O–H bond in the TS conformation (middle). In the product conformation (right), the catalytic acid (D221) has been reprotonated by proton transfer from nucleophilic water, to the bridge water, to the third water, and then to the carboxylic acid. Intermediate bond lengths are indicated in the TS by dashed black lines. Blue dotted lines indicate hydrogen bonding.

During system energy minimization and equilibration, the third water molecule spontaneously moved toward the active site from its initial position determined from the crystal structure with an aspartic acid at residue 175 (PDB ID 1QJW). The mutation from aspartic acid to alanine created a larger cavity that was easily able to accommodate the third water molecule, as shown in the upper-left panel of Fig. 8. The PDB ID ; 1HGW structure of the *Tr*Cel6A D175A also resolved a water molecule closer to the bridge water (3.4 Å). From the initial TS guesses, we performed AS and continued to obtain reactive trajectories as before. However, after proton transfer to the second (“bridge”) water, the proton then hopped to the third water, and then to D221. This longer water wire performed catalytic rescue and reprotonated the catalytic acid. Had our simulation set-up included additional water molecules in the QM region, we might have observed proton transfer to the bulk water, as our simulations and experimental structural studies^34^,^37^ indicate that bulk solvent is accessible from the active site even with the active site loop closed. Importantly, this simulation of the D175A mutant provides evidence for the potential of catalytic rescue by water. When residue 175 is part of the proton transfer mechanism, only two water molecules need to be aligned to allow proton hopping. Their alignment is aided by the S181 side chain and D401 backbone. In the case of the mutant, there must at least be a third water molecule properly aligned to form a water wire, which is a more rare event. Furthermore, previous studies have indicated that the presence of a negatively charged base, even separated by several water molecules, often accelerates proton dissociation from an acid.^90^ Previous studies have also demonstrated that longer water wires transfer protons at slower rates than shorter water wires.^90^–^92^ A recent QM/MM study of the inverting *O*-GlcNAc glycosyltransferase (OGT) calculated that the proton transfer to the catalytic base *via* a water molecule requires an activation energy 74% greater than direct transfer to α-phosphate, increasing the barrier from 23.5 kcal mol^–1^ to 41 kcal mol^–1^.^93^ Thus, our simulations provide the first results to support the hypothesis that a water wire can likely rescue catalytic activity at a lower rate than the WT due to the longer water wires involved, consistent with the lower residual activity observed for the D175A mutant.^34^

### Processivity

Beyond the hydrolytic mechanism, we are also keenly interested in understanding cellulose chain processivity. As described in the Computational methods, US was used to simulate processivity from the “pre-slide” to “slide” conformations, *i.e.* the advancement of a cellohexaose molecule into subsites –1 and –2 in the tunnel of *Tr*Cel6A with the active site loops in open position.

Completely unbiased simulations run for over 50 ns provided well-equilibrated structures for the processivity simulations. During simulations of the pre-slide conformation, all substrate glycosyl rings were observed only in the solution-stable ^4^C_1_ conformation. The same was true for the slide conformation simulations (both during the initial 50 ns of simulation and during an additional 250 ns of simulation) for all the substrate glycosyl rings except for the second glycosyl group (numbering from the leading monomer) which exclusively occupied the –1 binding site (Fig. 3). This subunit only adopted the ^2^S_O_/B_O,3_ conformation, which was also the reactant conformation observed in our hydrolysis studies and in crystal structures of cellulases trapped in the Michaelis complex (Fig. 7), indicating the importance of this subunit's ring distortion for reactivity.^3^,^89^ The active site loop remained in the open conformation throughout these unbiased simulations and during the biased simulations that followed.

The CV used for US was the RMSD of the leading cellobiose ring atom coordinates compared to a reference equilibrated conformation in the pre-slide conformation. “Pulling” (or “pushing”) the substrate from an equilibrated slide conformation toward (or further from) the pre-slide conformation, by biasing simulations along the CV, created initial configurations for each window.

**Fig. 9:**
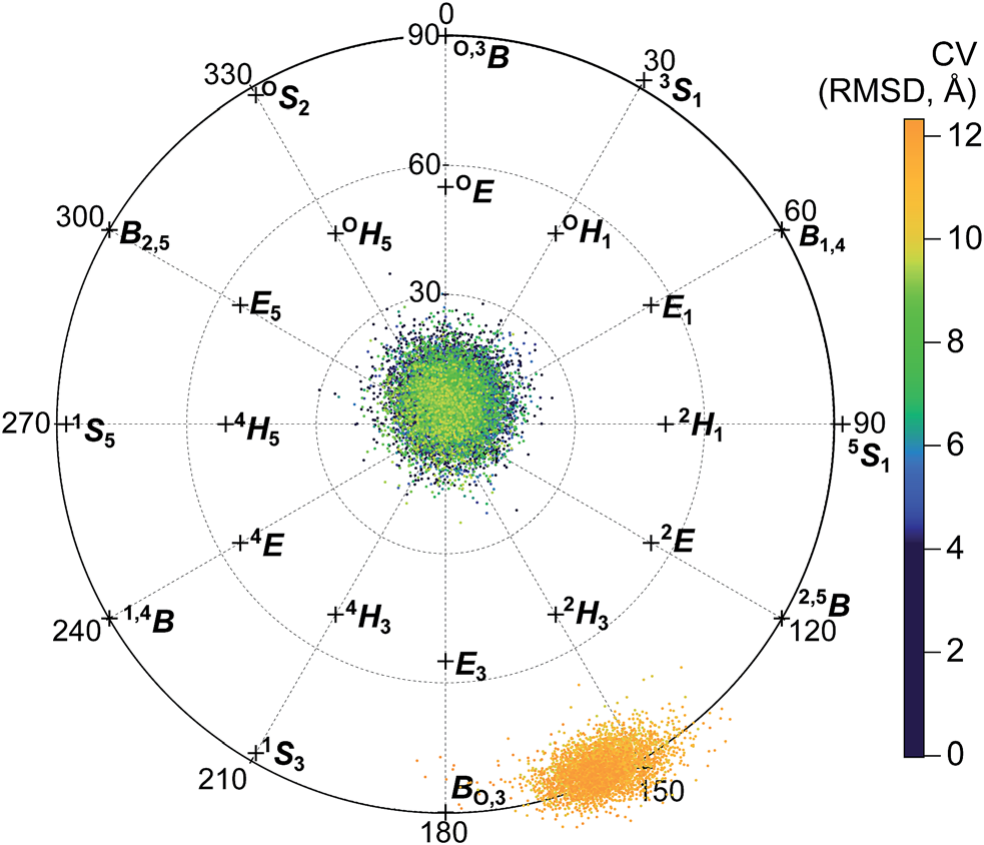
Projection of the Cremer–Pople puckering coordinates of the second glycosyl unit onto the northern hemisphere of the Cremer–Pople sphere. Each point represents a conformation collected during umbrella sampling (separated by 100 ps each), colored according to the corresponding value of the CV (RMSD) tracking procession into the active-site tunnel, described in the text.

An analysis of the puckering conformations revealed that all glycosyl units maintained the ^4^C_1_ conformation throughout the processivity simulations, except for the second glycosyl unit. This monomer displayed a distinct conformational change near the CV value of 9.62 Å. In this region, the second glycosyl unit perturbs from the ^4^C_1_ conformation to a ^2^S_O_/B_O,3_ conformation as it moves into the –1 position (Fig. 9). The ring did not freely move between the conformations in any of the windows, indicating that this conformational change was likely not well sampled. It is not surprising that a one-dimensional biasing coordinate was not sufficient to sample all important degrees of freedom for processivity. At least two more coordinates would be needed to correspond to the puckering coordinates shown in Fig. 9. Bias along three dimensions would be extremely costly, and the accuracy would be limited by level of accuracy of the Hamiltonian used. However, we can estimate the barrier for the puckering transition based on a previous study with a more accurate method: a QM study^89^ with CCSD(T)//B3LYP, *versus* the pure MM CHARMM force field used for the processivity simulations. This QM study estimated a barrier of approximately 6 kcal mol^–1^ for free glucose, without any stabilization by an associated enzyme.

**Fig. 10:**
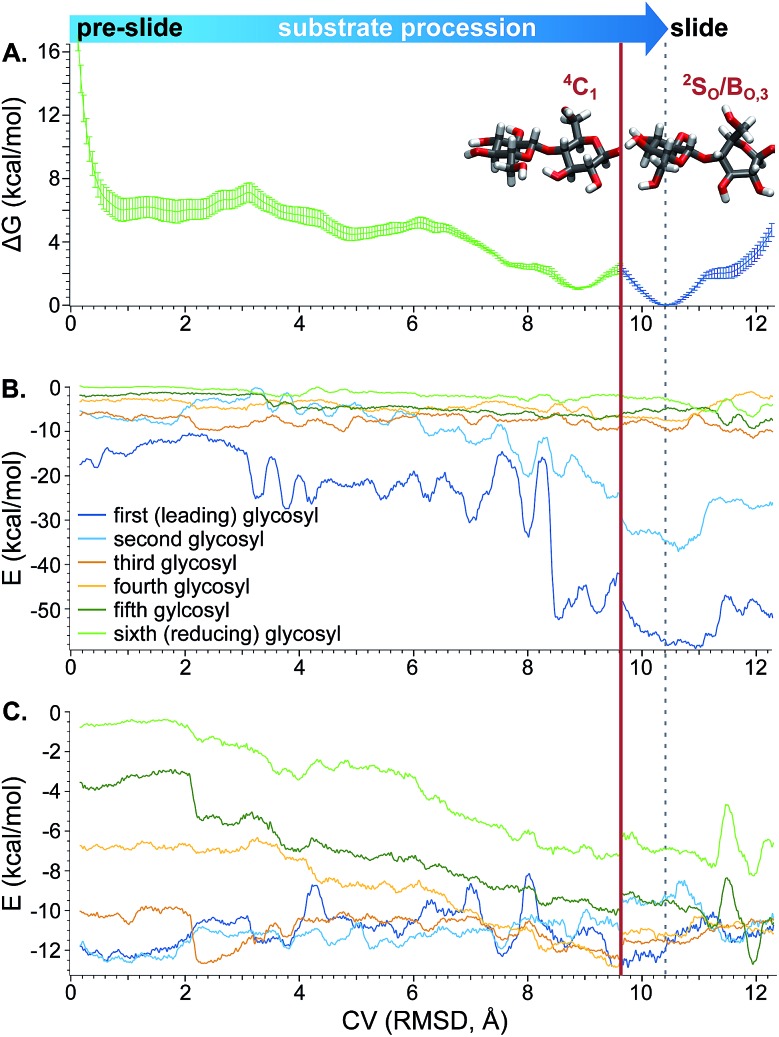
(A) PMF for procession from the “pre-slide” to “slide” conformation constructed from US and WHAM. (B) Electrostatic and (C) van der Waals interactions between each of the substrate glycosyl units and the enzyme along the processive CV, smoothed as described in the Computational methods. For all three plots, the *x*-axis represents the CV for procession through the active-site containing tunnel, as described in the text. The solid red line designates a puckering dividing line; to the left of the line, the second glycosyl unit is exclusively in the ^4^C_1_ conformation and to the right it is only in the ^2^S_O_/B_O,3_ conformation. The dotted gray line indicates the point along the CV corresponding to the slide conformation, at which point the substrate is in position in the active site for hydrolysis.

Therefore, a PMF generated based on biasing only the progress through the tunnel should be treated as an approximate pathway that does not include the full barrier corresponding to the puckering transition of the second glycosyl unit as it enters the –1 binding site. Still, analysis along this CV can be used to provide information about protein–sugar interactions in the tunnel. Thus, Fig. 10A shows the reconstructed PMF from WHAM constructed from separate analysis on (a) configurations in only the ^4^C_1_ conformation (to the left of the red line) and (b) configurations only along the Cremer–Pople equator in the ^2^S_O_/B_O,3_ conformation (to the right of the red line). The low-energy well at a CV value of approximately 10.4 Å corresponds to the “slide” position and the lowest values of the *x*-axis correspond to the pre-slide conformation. The values of the CV higher than 10.4 Å are not expected to be visited during the catalytic cycle, except due to energy fluctuations, but were explored to verify that the substrate would not normally process beyond the slide conformation. We found that if we biased the substrate to advance beyond a CV value of approximately 12.3 Å, the second glycosyl unit again adopted the ^4^C_1_ conformation as it was forced to leave the –1 position. The “pre-slide” snapshot in Fig. 3A was taken from near the end of the US simulations from a conformation with a CV value of 0.2 Å from the window centered at 0.0 Å. The “slide” snapshot in Fig. 3B was taken from near the end of the US simulation of the window centered at 10.5 Å (the CV value of that frame is 10.4 Å). The ESI† includes overlays of these two structures with their reference crystal structures.

The approximate PMF in Fig. 10A suggests that procession from the slide to pre-slide conformations occurs spontaneously. We used conformations generated during umbrella sampling to calculate the protein–sugar interactions shown in Fig. 10B and C. These unbiased interaction energies indicate that the low-energy well at the “slide” conformation is primarily due to strong electrostatic interactions between the protein and the leading cellobiose unit as it enters the product (–1 and –2) sites (Fig. 10B). As shown in the ESI,† the strongest of these electrostatic interactions involve residues that can hydrogen bond with hydroxyl groups of the substrate in the product sites (–1 and –2), occupied in the slide position: D137, at the end of the tunnel interacting with O3 at subsite –2; the side chain of D401 with O3 at –1; E399, also at the end of the tunnel, with O6 at –1; and K395 which hydrogen bonds with O3 at –1 and O6 at –2. These findings of hydrogen bonding between substrate and protein in the product site are consistent with previous studies suggesting that such interactions drive procession in CBH tunnels.^21^,^53^

Lower magnitude interactions with hydrophobic residues W269, W272, and W367 smoothly transfer between different glycosyl units along the processive path (see ESI†), increasing van der Waals interactions for the fourth, fifth, and sixth substrate glycosyl units as the substrate shifts into position for the next catalytic event (Fig. 10C). Such carbohydrate–aromatic residues have been shown to be important in processivity.^94^

Previous experimental studies indicated that Y169 is vital to puckering the glycosyl unit in the –1 position.^37^,^95^ We found van der Waals interaction and electrostatic interactions of only a few kcal mol^–1^ between Y169 and the first two glycosyl units, starting before the leading cellobiose unit occupies the product site (see ESI†). Previous computational studies have shown that W135, W269, and W367 are crucial for maintaining the ring conformation.^94^ All three residues show even stronger interactions with the substrate in the slide position compared to Y169.

## Conclusions

Here, we present the first atomic-level study of the dynamics of cellulose hydrolysis by the industrially important *Tr*Cel6A cellulase. The unbiased simulations provide evidence supporting the hypothesis that D175 can serve as the catalytic base *via* a bridging water molecule and highlight the importance of these water molecules in the reaction. Following the reaction path from the ensemble of collected TSs revealed that the glycosidic bond cleavage and transfer of the acidic hydrogen are almost complete before reaching the TS. Likelihood maximization identified that, of the 87 OPs tested, the key collective variables that best represent the RC are interatomic distances between the nucleophilic water oxygen and the anomeric carbon of the –1 glucopyranoside and between the nucleophilic water oxygen and the hydrogen that transfers to an adjacent water molecule, indicating that the nucleophilic attack drives the reaction. Determination of the PMF and transmission coefficient allowed calculation of the reaction barrier of 12.3 ± 0.4 kcal mol^–1^ and rate coefficient of 2.9 × 10^3^ s^–1^ at 300 K for this step. Hydrolysis thus has a larger barrier than our estimated processivity barrier (approximately 6 kcal mol^–1^). Previous computational^96^ and experimental^37^ studies have shown the inherent flexibility of the active site loop, with both the open and closed positions accessible at ambient conditions, indicating that loop opening and closing also have smaller transition barriers than hydrolysis. Thus, it is likely that hydrolysis is the rate-limiting step in the *Tr*Cel6A processive catalytic cycle. The calculated rate coefficient for this step is larger than the experimentally determined *k*_cat_ of 14 ± 2 s^–1^ for *Tr*Cel6A hydrolysis of cellohexaose,^34^ which may be due in part to inaccuracy of the semi-empirical method used to calculate the barrier.

Our simulations provide evidence consistent with the hypothesis that in the absence of a catalytic base, as in the D175A mutant, additional water molecules can perform catalytic rescue, shuttling the excess proton back to the catalytic acid (D221), as simulated, or potentially to the bulk water through a longer water wire. Ability for migration of water between the active site and bulk has been observed in our simulations. The role of *Tr*Cel6A D175 analogs in homologous enzymes has been investigated by activity studies of WT and mutant *Tf*Cel6A (formerly E2)^97^ and *Cellulomonas fimi* Cel6A (*Cf*Cel6A, formerly CenA).^98^ Alanine mutants of the analogous residues to *Tr*Cel6A D175, *Tf*Cel6A D79A and *Cf*Cel6A D216A, also show decreased activity, with the extent of decrease dependent on the substrate. The substrate-dependence on the rate of decrease suggests that the stereochemistry of residue is important. For example, we found that D175 in the *Tr*Cel6A WT hydrogen bonds with the second, “bridge” water, aiding in alignment of that molecule in a water wire. Additionally, the smaller side chain in *Tr*Cel6A D175A provided room for a third water molecule that could form a water wire with the two water molecules. The role of a second water molecule in WT hydrolysis raises the question of why the enzymes did not evolve a glutamic acid to serve as the catalytic base. It remains to be investigated whether the additional length would allow hydrolysis without the aid of a second water molecule. Even if that is possible, there is likely a reason why an aspartic acid is found at this position across homologous enzymes. The larger glutamic acid might interfere with substrate binding or movement into proper alignment in the active site. While no studies to our knowledge have reported a *Tr*Cel6A D175E mutant, activities of both *Tf*Cel6A D79E and *Cf*Cel6A D216E revealed that activity of such enzymes is decreased compared to WT. The residual activity of *Tf*Cel6A D79E is comparable to *Tf*Cel6A D79A for multiple substrates tested (phosphoric acid-swollen cellulose (SC), carboxymethyl-cellulose (SC), and filter paper).^97^ For *Cf*Cel6A, the D216E mutant activity is comparable to D216A for phosphoric acid-swollen cellulose, yet more than an order of magnitude lower on the non-native substrates 2′,4′-dinitrophenyl β-d-cellobioside and carboxymethyl-cellulose.^98^ Furthermore, the isosteric mutant *Tf*Cel6A D79N shows comparable activity to the D79A and D79E mutants, all reduced 2–3 orders of magnitude compared to WT, similar to the level of reduction for *Tr*Cel6A D175A compared to WT. Although D79N would not be able to accept a proton, its side chain can still participate in hydrogen bonding with water molecules to align them into a water wire. A mutation to a leucine would be interesting, as it is also isosteric but cannot participate in hydrogen bonding. Additionally, Vuong and Wilson studied the activity of the non-processive *endo*-glucanase *Tf*Cel6B WT and D226A mutant (homologous to *Tr*Cel6A D175A), which showed approximately one order of magnitude decrease in activity on bacterial microcrystalline cellulose, phosphoric acid-swollen cellulose, and phosphoric acid-treated cotton, yet a 10% increase in activity on carboxymethyl-cellulose.^39^ These experimental studies and the computational work reported here indicate that both reactivity and stereochemistry of the residue at this position is important for hydrolysis; hydrolysis can occur even without the residue accepting a proton, as long as a water wire is able to fill this role.

The key role of water molecules in the WT reaction and in the D175A mutant may explain why experiment alone was unable to definitively map the *Tr*Cel6A mechanism. Catalytic rescue by a water wire has been indicated in other enzymes as well, such as human carbonic anhydrase II.^91^ Like *Tr*Cel6A, carbonic anhydrase II shows residual activity when the primary proton acceptor is removed. Riccardi *et al.*'s investigation of mechanisms for catalytic rescue found that longer water wires required to reach an alternate stable state increased the barrier height for the reaction from 6.8 kcal mol^–1^ to 12.6 kcal mol^–1^ to 17.4 kcal mol^–1^ as the length of the water bridge was increased from two to three to four molecules, respectively.^91^ The finding that longer water wires allow proton transfer at slower rates than short water wires is supported by experimental and theoretical studies.^90^,^92^,^93^

We further investigated substrate processivity in *Tr*Cel6A. The simulations indicate that substrate movement into the product sites to be in position for the next catalytic event is a spontaneous reaction driven by multiple interactions with residues in the active site, primarily hydrogen bonding between charged side chains positioned to interact with the leading cellobiose unit when it occupies the –1 and –2 binding. The favorable interactions are consistent with previous studies of processive substrate motion and strong product inhibition.^3^,^21^,^53^ These simulations further reveal the all substrate glycosyl rings maintain the ^4^C_1_ conformation, except for the second-from-leading monomer. As this monomer moves into the –1 position, it distorts from the solution-stable ^4^C_1_ conformation to a ^2^S_O_/B_O,3_ pucker.

During hydrolysis, the –1 glycosyl ring changes more subtly, remaining on the equator of the Cremer–Pople sphere as it shifts from the ^2^S_O_ conformation in the reactant low-energy well toward the ^2,5^B conformation at the TS, and then relaxes toward the B_O,3_ conformation in the product low-energy well, with greater flexibility as the cleaved glycosidic bond no longer anchors the sugars as strongly in the –1 and –2 subsites. The functional importance of puckering for catalytic susceptibility activity has been previously studied,^89^ and puckering has been observed in the lowest-energy transition-state conformation even in non-enzymatic, thermochemical cleavage of cellulose's glycosidic bond.^99^ The energy barrier for non-enzymatic cleavage was calculated to be 54 kcal mol^–1^, more than four times greater than the barrier calculated here, indicating the significant effect achieved by multiple interactions between the protein and substrate.

This study delivers on the promise of computational studies to provide a basic understanding of the underlying enzymatic mechanisms.^100^–^103^ The studies presented here suggest two key features for inverting GH catalysis: (1) nucleophilic water molecule alignment to drive the reaction forward, and (2) water wires to shuttle the proton to the catalytic base. The important role of water in *Tr*Cel6A shown here may extend to inverting glycosyltranferases^59^ and other enzymes for which a catalytic base has been difficult to experimentally determine.

## Supplementary Material

Supplementary movie

Supplementary movie

Supplementary movie

Supplementary information
