# Supplementary material for: Who's on base? Revealing the catalytic mechanism of inverting family 6 glycoside hydrolases
Source: Chem Sci. 2016 Jun 1;7(9):5955–68. doi: 10.1039/c6sc00571c (PMC6091422; doi:10.1039/c6sc00571c)
Supplement: Supplementary file 4 [file SC-007-C6SC00571C-s004.pdf]

Electronic Supplementary Material (ESI) for Chemical Science.
This journal is © The Royal Society of Chemistry 2016

Electronic Supplementary Information (ESI) for:

Who's on base? Revealing the catalytic mechanism of inverting Family 6 glycoside hydrolases

Heather B. Mayes,[a,b] Brandon C. Knott,[b] Michael F. Crowley,[b]
Linda J. Broadbelt,[a] Jerry Ståhlberg,[*c] and Gregg T. Beckham[*b]

Detailed Computational Procedures

Enzyme System Configuration

For all starting conformations, disulfide bridges were constructed between Cys176/Cys235 and between Cys368/Cys415. The residues Asp221, Glu107, Asp170, and Asp419 were protonated. The system was then solvated with water to a 75 Å cube, with five sodium ions added to neutralize the system charge. The total number of atoms in the Michaelis complex, pre-slide, and slide conformations were 42,886, 42,883, and 42,886 atoms, respectively.

The CHARMM36 force field was used for all simulations and all parts of the system: the enzyme,[1–3] carbohydrate ligands,[4,5] and sodium ions[6] with the TIP3P model for water.[7]

As noted in the manuscript, the CHARMM[8] package was used to build and equilibrate all systems. Each of these solvated complexes was first minimized using CHARMM with a non-bonded cutoff distance of 12 Å with a switching distance of 10 Å and a non-bonded pair list distance of 16 Å. The Particle Mesh Ewald method[9] was used with a Gaussian distribution width of 0.34 Å sixth order b-spline, and 80 grid points in each dimension. The systems were minimized by alternating between 250 steps of steepest-descent and adopted basis Newton-Raphson for a total of 2,500 steps. Next, the systems were heated from 100 K to 300 K in 50-K increments over 1 ns in the NVT ensemble. The remaining procedures for the two types of simulations are detailed below. Unless otherwise noted, after the initial equilibration and heating in CHARMM, all remaining simulations were performed using the Amber[10,11] software package version 12, in the NPT ensemble using the Andersen thermostat[12] set to 300 K and isotropically controlling the pressure to 1.0 bar with a relaxation time of 2.0 ps. Velocities were randomized every 1000 MD steps. QM/MM simulations utilized a 1 fs timestep while MM-only simulations utilized a 2 fs timestep. The CHAMBER13[13] program was used to convert the CHARMM protein structure files, coordinate files, and force field files to Amber topology file and coordinate files.

WT Hydrolysis

As discussed in the manuscript Introduction, we used AS to collect an ensemble of transition states for glycosidic cleavage by hydrolysis. An initial trajectory connecting a reactant basin (A) to a product basin (B) is required to begin transition path sampling. The Hamiltonian used to generate the initial path can be biased, provided that equilibration runs are completed before data is collected.[14–16] In this section, we describe how we modeled the QM region, minimized and equilibrated the system, used targeted MD to generate candidate conformations between the reactant and product basins, performed AS to collect an ensemble of transition states, used that data to chose a reaction coordinate based on Likelihood Maximization, and used the reaction coordinate to guide Equilibrium Path Sampling (EPS) to chart the PMF for the reaction. Details on the procedure for each of these steps are provided below.

QM Region

QM/MM simulations were used to model the bond-breaking and formation events. The QM region is shown in Fig. S1. The components were chosen to include residues theorized to be vital to hydrolysis, a subsection of the substrate, and the two water molecules resolved from crystal studies.[17,18] Specifically: the side chains of residues D175, D221, S181, and Y169 are included, excluding the C^{α} atoms which were replaced by hydrogen atoms. Only the D401 backbone C=O fragment, capped with hydrogen atoms, is included. While the MM region includes a cellohexaose substrate, the QM region includes a cellobiose unit (capped by hydrogen atoms) in the −1 and +1 binding positions, capturing the glycosidic bond to be hydrolyzed. The overall charge of the QM region is −1.

As previously noted, the molecular mechanics force fields employed were TIP3P for water and CHARMM36 for all other components. The QM region was modeled with the self-consistent

[a] Department of Chemical and Biological Engineering, Northwestern University, Evanston, IL 60208, United States
[b] National Bioenergy Center, National Renewable Energy Laboratory, Golden, CO 80401, United States
[c] Department of Chemistry and Biotechnology, Swedish University of Agricultural Sciences, SE-75007, Uppsala, Sweden
* E-mail: jerry.stahlberg@slu.se, gregg.beckham@nrel.gov

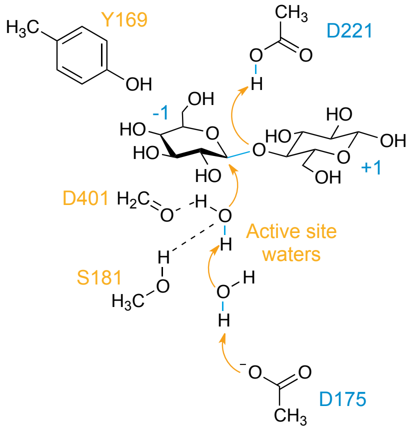

Fig. S1 A two-dimensional representation of the atoms included in the QM region of the QM/MM simulations, showing the capping hydrogen atoms on the portions of protein residues included in the region. Orange arrows indicate which bonds will be formed and blue lines indicate which bonds will be cleaved. The dashed black lines reflect hydrogen bonding between the D401 backbone, nucleophilic water, and S181 side chain. This scheme further reflects that the -1 glucopyranoside ring is puckered to the $^{2,5}B$ conformation in the reactant conformation due to steric interactions with Y169.

charge density functional tight-binding (SCC-DFTB) method with second-order terms in the charge density fluctuations,[19,20] which is referred to as SCC-DFTB2 to distinguish it from the implementation with third-order corrections (SCC-DFTB3).[21] The SCC-DFTB methods are approximate methods based on DFT calculations, in contrast with traditional semi-empirical methods such as AM1 and PM6, which are based on Hartree-Fock theory using empirical data from a particular chemical environment.[22] SCC-DFTB has become a popular choice for modeling biomolecules, as it provides the computational efficiency required to capture the time and length scales necessary to investigate biologically important phenomena, while providing greater accuracy than the traditional semi-empirical methods. Improvements include a more reliable description of weak-binding forces, reaction energies, geometries, and frequencies, benchmarked against biomolecules such as peptides and DNA fragments, often used in combination with a CHARMM force field.[19,22–26] SCC-DFTB has known limitations, particularly evident in describing excess protons in bulk water, such as overestimating the number of solvation shell water molecules coordinating with excess protons.[27,28] The QM region in our system includes only the active site, and thus our results are not expected to be tainted by the shortcomings of modeling bulk water. Interestingly, the SCC-DFTB2 method implemented in Amber has yielded proton transfer barriers similar to Car-Parrinello molecular dynamics simulations using the BLYP functional,[29] providing further confidence that our methodology allows meaningful mechanistic analysis for proton transfer mechanisms in the enzyme active site.[30]

These studies indicate that SCC-DFTB2 is well suited for the purpose of our current work: to allow extensive sampling of a highly multidimensional energy landscape that can uncover a complex reaction path that includes proton transfer events. Our primary goal is to determine the mechanism; precisely calculating final energetics is a secondary concern. Computational determination of the *Tr*Cel7A hydrolysis rate with the same procedures used[31] show remarkable agreement with experimental rates recently published,[32] providing confidence in our choice for computational methods in this work.

Minimization and Equilibration

We found that even in short (100 ps) simulations, water molecules observed in the active site (see Fig. 2 in the manuscript) exchanged with other nearby water molecules also identified within the experimental crystal structure. Keeping the identity of these two water molecules consistent across many simulations would provide simplicity without biasing the results. Thus, their positions were restrained during these equilibration and minimization steps, each of which consisted of 2500 cycles. First, only the added water molecules and ions were unrestrained during minimization with MM only, with the rest of the system held fixed by Cartesian restraints with a high force constant (10,000 kcal/mol-Å^2). Then, all but the ligand and noted active site waters were unrestrained during an MM-only minimization. A final MM-only minimization was performed with the entire system unrestrained. This was followed by a 100 ps MM-only equilibration run. To prevent the active site's water molecules from exchanging with nearby waters, restraints were enforced on the distances shown in Scheme S2, in the shape of a flat-bottomed-well with a force constant of 25 kcal/mol-Å^2 in the parabolic region. The QM region was then defined and a QM/MM minimization of 2500 cycles was completed without any restraints. A 100 ps QM/MM equilibration step was then completed using the settings and constraints as used during the MM equilibration. Longer equilibration simulations were not run to prevent moving far from the Michaelis complex captured in the crystal structure.

Generating TS Guesses for AS

The one-step inverting pathway requires multiple bond rearrangements. The hypothesis that D175 acts as the catalytic base mediated by a bridging water requires four new bonds to be formed (orange arrows in Fig. S1) and four bonds to be cleaved (light blue bonds in Fig. S1). To look for conformations near a transition state, we generated a series of guesses by using targeted MD to set intermediate distances for these eight bond rearrangements. Forty-eight guesses were generated by sampling from the C-O bond distances of 2.1, 2.2, 2.3, or 2.3 Å, and O-H bond distances were set to 1.3, 1.4, 1.5, or 1.6 Å. These guesses were created from the QM/MM equilibrated conformation by

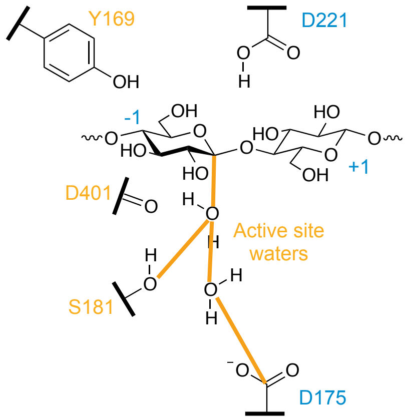

Fig. S2 The bold orange lines indicate where restraints were added to prevent the active site waters from exchanging with nearby waters during the *Tr*Cel6A WT equilibration.

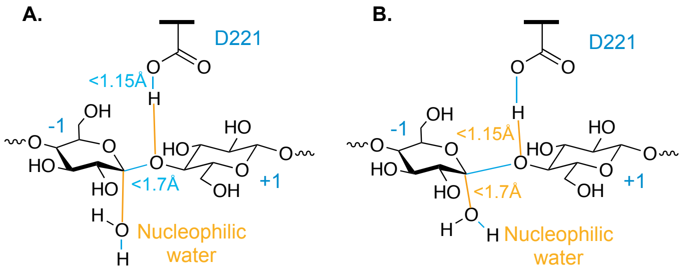

Fig. S3 A. The end of a forward or backward AS trajectory is identified as visiting the reactant basin if the distance between the acidic hydrogen and the D221 carboxylic acid is less than 1.15 Å and the glycosidic bond remained intact (defined by a distance of less than 1.7 Å). B. The endpoint of an AS trajectory is identified as visiting the product basin if the distance between the acidic hydrogen from D221 and the +1 glucopyranoside O4 is less than 1.15 Å and the nucleophilic water oxygen is less than 1.7 Å from the -1 glucopyranoside C1.

changing the eight distances from the initial to target distance in 20 steps, adjusting the distance linearly across the steps using a flat-bottomed potential with a force constant of 450 kcal/mol-Å^2 in the parabolic region. Each step was run for 2 ps.

To determine whether any of these guesses would produce a reactive trajectory, the guesses were used as seeds for AS runs of 50 trajectories each. Per the AS procedure, each trajectory began by generating random velocities from the Boltzmann distribution. An unbiased trajectory in this "forward" direction was first followed for a δt of 0.025 ps (25 MD steps) to save the coordinates for potential use in the next trajectory, and then followed for another 750 steps to obtain an endpoint geometry for analysis. Additionally, the generated velocities were reversed so the trajectory could also be followed in the "backward" direction for 750 steps to obtain the other endpoint. The following distances were used to determine whether the endpoint in each trajectory visited the reactant or product basin (Fig. S3): D221 O$^{\delta 2}$ (carboxylic oxygen) to D221 H$^{\delta 2}$ (acidic hydrogen); D221 H$^{\delta 2}$ (acidic hydrogen) to $-1/+1$ glycosidic oxygen; $-1/+1$ glycosidic oxygen to -1 C1; and -1 anomeric carbon to nucleophilic water O. If one of the two endpoints visited the reactant basin and the other visited the product basin, the trajectory was identified as reactive or "accepted". We also tracked whether both endpoints visited the reactant basin or both visited the product basin. If the distance criteria did not align with one of those three options, the trajectory was determined to be inconclusive.

Of the 48 TS guesses serving as seeds for AS runs, 37 yielded at least one reactive trajectory of the 50 trajectories run. Seven AS runs had an acceptance rate greater than 30 %, which is reasonable for this Monte Carlo type algorithm.[33] Several accepted trajectories were checked in detail to verify the distance requirements used to classify visiting the reactant or product basin. We found that the endpoints reflected all eight bond rearrangements indicated in Fig. S1. Of the rejected trajectories, we found that some seed endpoints more often visited the reactant basin while other seed endpoints more often visited the product basin. This provided confidence that the range of C-O and O-H distances used to generate TS guesses spanned a range of potentially early and late transition states.

Aimless Shooting and Likelihood Maximization

We used endpoints of the AS runs to test our TS guesses as seeds for production AS production runs to explore the ensemble of TSs. We selected the five endpoints from the five runs with the highest acceptance ratios. Each endpoint was used to seed ten AS production runs, for a total of 50 AS production runs. Each production run consisted of a series of 2500 trajectories. Unlike enhanced sampling methods such as umbrella sampling[34] and metadynamics,[35] the forces on the system are not perturbed to increase sampling of high-energy regions of the potential surface. Thus, these trajectories are not biased according to *a priori* assumptions about which degrees of freedom (collective variables) best define the transition state.[14,36]

As in the AS runs used to test the TS guesses, a δt of 0.025 ps was used to obtain potential alternate starting points. The forward and backward trajectories were run for 750 steps each to obtain endpoints to determine whether they visited the reactant or product basins, using the same distance criteria previously described. Data from 32 of the 50 AS production runs were analyzed; the other runs were stopped due to the aimless exploration of the transition state ensemble leading the runs into regions when QM convergence was slow and/or acceptance rates became low (less than 6 %). Furthermore, the data from the first 500 trajectories in each AS run were not used to allow equilibra-

tion/decorrelation from the biased, targeted MD runs used to create the initial TS guesses. Thus, 64,000 AS shooting points were used for the next step, Likelihood Maximization. This method makes use of the data collected during the AS simulations. Saved geometries of the shooting points are used to calculate the values of a postulated set of order parameters (OPs). The values of the OPs at each point are tested to determine if there is correlation with changes in those variables and whether the trajectory generated from that point was reactive, both endpoints went to the reactant basin, or both to the product basin. Thus, the OPs are searched for candidate reaction coordinates.

We chose a list of candidate OPs based on analysis of selected reactive trajectories to identify substrate and enzyme conformational changes along the trajectory. We additionally gathered proposed order parameters suggested in previous research on *Tr*Cel6A.[18] The OPs can take a variety of forms. The set of candidate OPs includes distances between atom centers, differences of distances, angles, dihedral angles, and Cremer-Pople ring puckering coordinates. The full set of tested OPs are listed in Table S1. The Likelihood Maximization results from the 32,000 AS points (32 runs using points from trajectories 501 to 1500) are reported. Following forward and backward trajectories from these points resulted in 6,910 accepted (reactive) trajectories; 17,121 trajectories with both endpoints in the A (reactant) basin; 7,071 trajectories with both end points in the B (product) basin; and 898 inconclusive trajectories.

Committor Analysis

The committor probability, $p_B(\mathbf{x})$, denotes the fraction of trajectories initiated from a configuration $\mathbf{x}$ with momenta randomly chosen from the Boltzmann distribution that will visit (commit to) the product basin B. Transition states are identified by $p_B(\mathbf{x}) = 0.5$. The Likelihood Maximization calculates optimal parameters to center $p_B(\mathbf{x}) = 0.5$ on an RC value of 0.5. To test if the putative RCs identified by Likelihood Maximization accurately describe the reaction coordinate, we generated a series of configurations with a calculated RC value near zero (abs(RC($\mathbf{x}$)) <0.01). We looked for AS points likely to be good TS candidates as defined by A) abs(RC($\mathbf{x}$)) <0.1, and B) a minimum 50 % acceptance rate for the ten trajectories run before and after the point ($\mathbf{x}$). From these 30 points, we initiated 30 EPS runs to collect 91 decorrelated configurations with abs(RC($\mathbf{x}$)) <0.01, using six nodes per string, three MD steps separating each node, and a window of abs(RC($\mathbf{x}$)) <0.03. The first and then every twentieth harvested point within abs(RC($\mathbf{x}$)) <0.01 (for a total of 300 shooters) were then used to shoot 20 forward trajectories, each drawing fresh momenta from the Boltzmann distribution. The resulting data on which basin the endpoint committed to was used to create p_B histograms.

Equilibrium Path Sampling

We performed five EPS runs to gather data for our hydrolysis PMF. To generate seeds for these separate runs, we wished to sample from different regions of the transition state ensemble. Thus, we selected one AS shooting point from five different runs which had acceptance rates of at least 20 %. In pursuit of highly decorrelated points, we chose points from no earlier than the 2000 of the 2500 trajectories from each run, choosing an accepted point from within a region with a high acceptance rate. From each of these five points, we collected accepted trajectories saving the configuration of each step for 750 steps in the “forward” direction and also reversed trajectories for the “backward” direction, calculating the values of the OPs and the RC from Likelihood Maximization at every step. Based on the range of the RC values for these runs, we chose to set up 25 EPS windows ranging from -8 to 6, with each window sharing 10 % overlap on each side. 10,000 strings were run in each window, with each string containing six nodes separated by three MD timesteps each (3 fs). The relative free energy in each window is calculated from the equilibrium probability distributions of visiting different regions of the RC within the window, separated into 0.06 Å bins. A PMF for the full RC range is obtained by matching free energies in the overlapping portions of each window.[37,38]

Determination of -1 Glucopyranoside Puckering Conformations

The transition state conformations were harvested from the AS points that led to accepted trajectories. As described in the methods for Likelihood maximization, these points came from 32 independent runs, analyzing data from the trajectories 501 to 1500. The conformations for reactant or product were obtained by running unconstrained QM/MM simulations to harvest 2000 points separated by 0.5 ps each, gathered from five independent simulations started from the endpoints of the trajectories chosen to seed the EPS runs, after first equilibrating for 1 ps. This short equilibration run was determined to be sufficient to ensure that the sampled conformations were in the hydrolysis reactant or product basins.

Mutant Hydrolysis Simulations

For one set of *Tr*Cel6A D175A hydrolysis simulations, the QM region for the mutant was identical to the region used for the WT except for the omission of residue 175, leaving the overall charge of the QM region neutral. The results of these simulations are discussed only here in the ESI. The minimization and equilibration steps were identical, except only three restraints were used to keep the active site waters from exchanging with nearby waters, omitting the fourth restraint involving residue 175, shown in Fig. S2. Initial TS guesses were generated by using the same targeted MD strategy, with six target distances set instead of eight,

Table S1 Order parameters tested as candidate reaction coordinates for the *Tr*Cel6A hydrolysis reaction.

OP index	parameter type	description
1	distance	D221 $O^{\delta 2}$ (carboxylic oxygen) to D221 $H^{\delta 2}$ (acidic hydrogen)
2	distance	D221 $H^{\delta 2}$ (acidic hydrogen) to $-1/+1$ glycosidic oxygen
3	distance	$-1/+1$ glycosidic oxygen to -1 C1
4	distance	-1 anomeric carbon to nucleophilic water O
5	distance	nucleophilic water O to nucleophilic water transferring proton H2
6	distance	nucleophilic water transferring proton H2 to bridge water O
7	distance	bridge water O to bridge water transferring proton H2
8	distance	bridge water transferring proton H2 to D175 $O^{\delta 2}$ (carboxylic oxygen)
9	difference	OP2 - OP1
10	difference	OP3 - OP2
11	difference	OP4 - OP3
12	difference	OP5 - OP4
13	difference	OP6 - OP5
14	difference	OP7 - OP6
15	difference	OP8 - OP7
16	pucker	Cremer-Pople ϕ for -1 glucopyranoside
17	pucker	Cremer-Pople θ for -1 glucopyranoside
18	pucker	Cremer-Pople Q for -1 glucopyranoside
19	pucker	Cremer-Pople ϕ for $+1$ glucopyranoside
20	pucker	Cremer-Pople θ for $+1$ glucopyranoside
21	pucker	Cremer-Pople Q for $+1$ glucopyranoside
22	dihedral	D221 C^{α} to D221 C^{β} to D221 C^{γ} to D221 $O^{\delta 2}$
23	dihedral	S181 C^{α} to S181 C^{β} to S181 O^{γ} to S181 H^{γ}
24	dihedral	S181 C^{β} to S181 O^{γ} to S181 H^{γ} to nucleophilic O
25	dihedral	D175 C^{α} to D175 C^{β} to D175 C^{γ} to D175 $O^{\delta 2}$
26	dihedral	-1 glucopyranoside C5 to O5 to C1 to $-1/+1$ glycosidic O
27	dihedral	-1 glucopyranoside C3 to C2 to C1 to $-1/+1$ glycosidic O
28	dihedral	-1 glucopyranoside C5 to O5 to C1 to nucleophilic water O
29	dihedral	-1 glucopyranoside C3 to C2 to C1 to nucleophilic water O
30	angle	S181 O^{γ} to S181 H^{γ} to nucleophilic water O
31	angle	D401 C (backbone) to D401 O (backbone) to nucleophilic water H1
32	distance	S181 H^{γ} to nucleophilic water O
33	distance	D401 O (backbone) to nucleophilic water H1
34	distance	nucleophilic water O to nucleophilic water non-transferring proton H1
35	distance	bridge water O to bridge water non-transferring proton H1
36	distance	C1 to O5 of -1 glucopyranoside residue
37	distance	$+1$ glucopyranoside O3 to bridge water non-transferring proton H1
38	distance	Y169 OH to -1 glucopyranoside O5
39	distance	Y169 OH to -1 glucopyranoside C5
40	distance	Y169 OH to -1 glucopyranoside C6
41	distance	Y169 OH to -1 glucopyranoside O6
42	distance	$-1/+1$ glycosidic oxygen to $+1$ C4
43	angle	-1 anomeric carbon to $-1/+1$ glycosidic oxygen to $+1$ C4
44	dihedral	-1 glucopyranoside O5 to C1 to $-1/+1$ glycosidic O to $+1$ C4

Continued on next page

Table S1 continued.

OP index	parameter type	description
45	dihedral	-1 glucopyranoside C1 to $-1/+1$ glycosidic O to $+1$ C4 to C5
46	dihedral	-1 glucopyranoside O5 to C5 to C6 to O6
47	dihedral	-1 glucopyranoside C4 to C5 to C6 to O6
48	dihedral	-1 glucopyranoside C5 to C6 to O6 to HO6
49	dihedral	$+1$ glucopyranoside O5 to C5 to C6 to O6
50	dihedral	$+1$ glucopyranoside C4 to C5 to C6 to O6
51	dihedral	$+1$ glucopyranoside C5 to C6 to O6 to HO6
52	distance	-1 O6 to D221 $H^{\delta 2}$ (acidic hydrogen)
53	distance	-1 HO6 to D221 $O^{\delta 1}$ (carboxylic oxygen)
54	distance	-1 HO6 to D221 $O^{\delta 2}$ (carboxylic oxygen)
55	distance	-1 HO6 to $-1/+1$ glycosidic oxygen
56	distance	$+1$ O6 to D221 $H^{\delta 2}$ (acidic hydrogen)
57	distance	$+1$ HO6 to D221 $O^{\delta 1}$ (carboxylic oxygen)
58	distance	$+1$ HO6 to D221 $O^{\delta 2}$ (carboxylic oxygen)
59	distance	$+1$ HO6 to $-1/+1$ glycosidic oxygen
60	distance	$+1$ HO3 to D175 $O^{\delta 1}$
61	distance	$+1$ HO2 to D175 $O^{\delta 1}$
62	distance	D221 $H^{\delta 2}$ (acidic hydrogen) to D175 $O^{\delta 1}$
63	distance	D221 $H^{\delta 2}$ (acidic hydrogen) to D175 $O^{\delta 2}$
64	dihedral	D175 C to C^{α} to C^{β} to C^{γ}
65	dihedral	S181 C to C^{α} to C^{β} to O^{γ}
66	dihedral	D221 C to C^{α} to C^{β} to C^{γ}
67	dihedral	Y169 C to C^{α} to C^{β} to C^{γ}
68	dihedral	Y169 C^{α} to C^{β} to C^{γ} to $C^{\delta 1}$
69	dihedral	E399 C^{α} to C^{β} to C^{γ} to C^{δ}
70	dihedral	E399 C^{β} to C^{γ} to C^{δ} to $O^{\varepsilon 1}$
71	dihedral	D401 C^{α} to C^{β} to C^{γ} to $O^{\delta 1}$
72	dihedral	D405 C^{α} to C^{β} to C^{γ} to $O^{\delta 1}$
73	dihedral	W135 C to C^{α} to C^{β} to C^{γ}
74	dihedral	W135 C^{α} to C^{β} to C^{γ} to $C^{\delta 1}$
75	dihedral	W269 C to C^{α} to C^{β} to C^{γ}
76	dihedral	W269 C^{α} to C^{β} to C^{γ} to $C^{\delta 1}$
77	dihedral	W367 C to C^{α} to C^{β} to C^{γ}
78	dihedral	W367 C^{α} to C^{β} to C^{γ} to $C^{\delta 1}$
79	distance	E399 $O^{\varepsilon 1}$ to -2 glucopyranoside C4
80	distance	W135 $C^{\varepsilon 2}$ to -2 glucopyranoside C6
81	distance	W135 $C^{\varepsilon 3}$ to -2 glucopyranoside C5
82	distance	W269 $C^{\zeta 3}$ to $+2$ glucopyranoside O5
83	distance	W269 $C^{\zeta 3}$ to $+2$ glucopyranoside C5
84	distance	W269 $C^{\zeta 3}$ to $+2$ glucopyranoside C6
85	distance	W367 $N^{\varepsilon 1}$ to $+1$ glucopyranoside C2
86	distance	D401 O (backbone) to nucleophilic water O
87	angle	D401 O (backbone) to nucleophilic water H1 to nucleophilic water O

as two of the original eight involved residue 175. Distances for 25 initial guesses were chosen to match distances used in the WT TS guesses with the highest resulting acceptance rates. Another 26 guesses were generated to match distances from accepted WT AS shooting points near the ends of AS runs with high acceptance percentages. The 51 TS guesses for the *Tr*Cel6A D175A mutant were used as seeds for AS runs of 50 trajectories each.

A second set of *Tr*Cel6A D175A hydrolysis simulations was completed including a third water molecule in the QM region. The results of these simulations are described in the main text. As with the other two water molecules in the QM region, the third water molecule was observed in the 1QJW crystal structure. The molecule chosen for the third water had a distance of 3.4 Å between its oxygen and that of the second, "bridge" water oxygen. During minimization and equilibration, a weak forth restraint was included for the third water molecule with a wide flat-bottomed potential to allow it to stay in its original position identified in the crystal structure or move closer into the active site, with no bias penalty for either option. TS guesses were generated based on distances from accepted WT AS shooting points near the ends of AS runs with high acceptance percentages.

Production AS runs, Likelihood Maximization, committor analysis, and EPS simulations were not completed for the mutant enzyme.

Unrestrained MM-only simulations were run in NAMD[39] starting from the minimized and equilibrated mutant structure. These were conducted in the NPT at 300 K and 1.0 atm with the SHAKE algorithm used to fix all hydrogen distances.[40] A nonbonded cutoff distance of 10 Å, with a switching distance of 9 Å, and a nonbonded pair list distance of 13 Å was used. The Particle Mesh Ewald method was used to describe the long-range electrostatics[9] with a sixth order b-spline, a Gaussian distribution with a width of 0.312 Å, and 1 Å grid spacing. The velocity Verlet multiple time-stepping integration scheme was used evaluating the full nonbonded interactions every 2 timesteps, the full electrostatics interactions every 4 timesteps, and 20 timesteps between atom reassignments.

Processivity Simulations

*Tr*Cel6A processivity was investigated using umbrella sampling (US)[34] and employed the weighted histogram analysis method (WHAM)[41,42] to produce the PMF. As previously noted, initial configurations for the "slide" and "pre-slide" (Figure 3 in the main text) configurations were initially build, minimized, and heated using CHARMM. Unrestrained equilibration runs for each configuration were in NAMD[39] for over 50 ns, with the same parameters noted for the NAMD simulations for Mutant Hydrolysis Simulations. The endpoint structures from these runs were aligned using VMD's RMSD trajectory tool[43] for use as starting points in the processivity simulations (later, an additional 250 ns of simulation were completed for the slide conformation to observe stability of the active site loop). The "slide" configuration, in which the product sites are occupied, was used as the reference state for umbrella sampling simulations performed with Amber 12's "targeted MD" utility. As in a the recent work on *Tr*Cel7A,[44] the coordinate for umbrella sampling path was the RSMD between the reference state and active simulation non-hydrogen atoms of the leading cellobiose unit. The initial RMSD between the "slide" and "pre-slide" configurations was 10.5 Å. We defined windows for umbrella sampling separated by 0.25 Å, ranging from 0 to 12.5 Å. To create initial configurations for each window, the leading cellobiose unit was pulled to the desired RMSD over 5000 steps using a force constant of 30.0 kcal/mol-Å^2 to the desired RMSD value. To prevent the protein moving along with the substrate, it was initially constrained with a larger harmonic force constant of 200 kcal/mol-Å^2. Umbrella sampling production runs were simulated for 40 ns with a harmonic force constant of 5.0 kcal/mol-Å^2 on the leading cellobiose non-hydrogen atoms, discarding the data from the first 2 ns to allow for equilibration. The protein was left mostly unconstrained to allow natural dynamic responses to the substrate; to prevent the whole protein from moving to allow the substrate to occupy the low-energy wells at the "slide" and "pre-slide" positions, a 20.0 kcal/mol-Å^2 restraint was placed on seven backbone C^α atoms of residues S106, A150, D200, N247, A280, I330, and Q437. These residues were chosen based on a previous study that demonstrated that they show a low root mean square fluctuation[45] and because they were located at a variety of locations on the enzyme but not near the active site tunnel.

Separate PMFs for procession were calculated with a) the second glycosyl unit in the $^4C_1$ conformation, and b) the second glycosyl unit in the $^2S_O/B_{O,3}$ conformation. Data from windows centered at 0.00 through 9.50Å were used for the PMF for the $^4C_1$ conformation, while data from windows 9.75 through 12.25Å were used to create the PMF fro the $^2S_O/B_{O,3}$ conformation, with the PMFs aligned at 9.625Å as indicated by the red line on the composite PMF. Histograms of processivity CV values for each window were checked for overlap (Figure S4) and sampling in all regions of the CV before calculating the PMFs. The WHAM bin spacing was 0.05 Å and used a tolerance of $1x10^{-9}$, with both parameters chosen based on convergence of the PMF. Data from the last 38 ns of the 40 ns simulations in each window were divided into four blocks. The reported PMF reflects the average and standard error from calculating the PMF on each block.

Additional Results and Discussion

WT Hydrolysis

To better show the range of puckering conformations sampled by the reactant, TS, and product, Fig. S5 shows the same data as in Fig. 7 in the main text, with each state shown on a separate graph so no data is obscured.

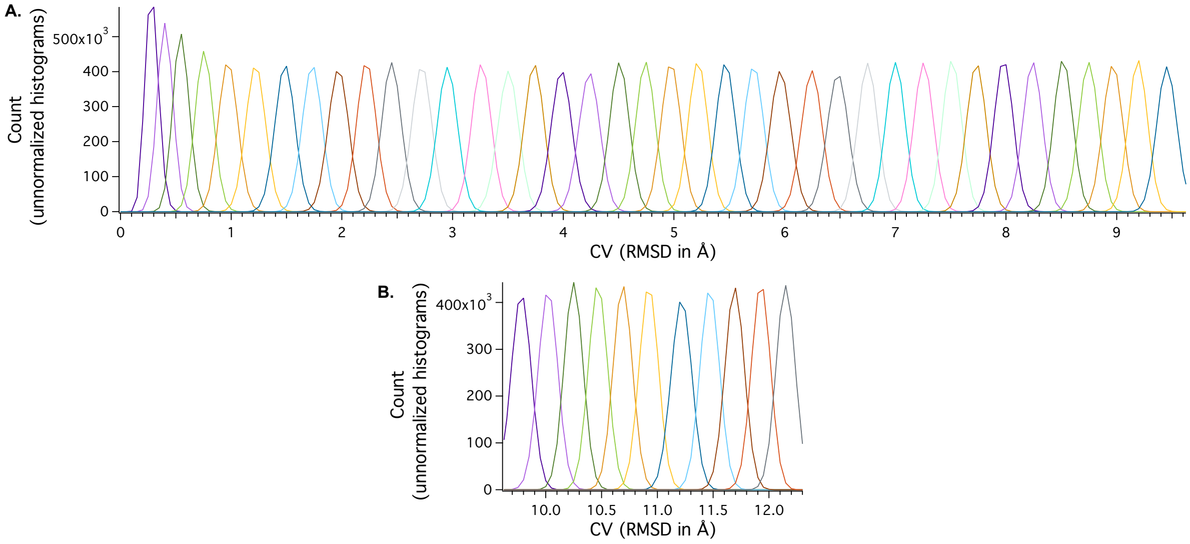

Fig. S4 Histogram overlap from the umbrella sampling windows used to generate the PMFs for processivity. A) Data from windows centered at CV values of 0.00 through 9.50Å (0.25 Å window spacing) were used to generate a PMF for procession with all substrate subunits in the $^4C_1$ conformation. B) Data from windows centered at 9.75 through 12.25Å were used to generate the PMF for procession with the second substrate monomer in the $^2S_O/B_{O,3}$ conformation.

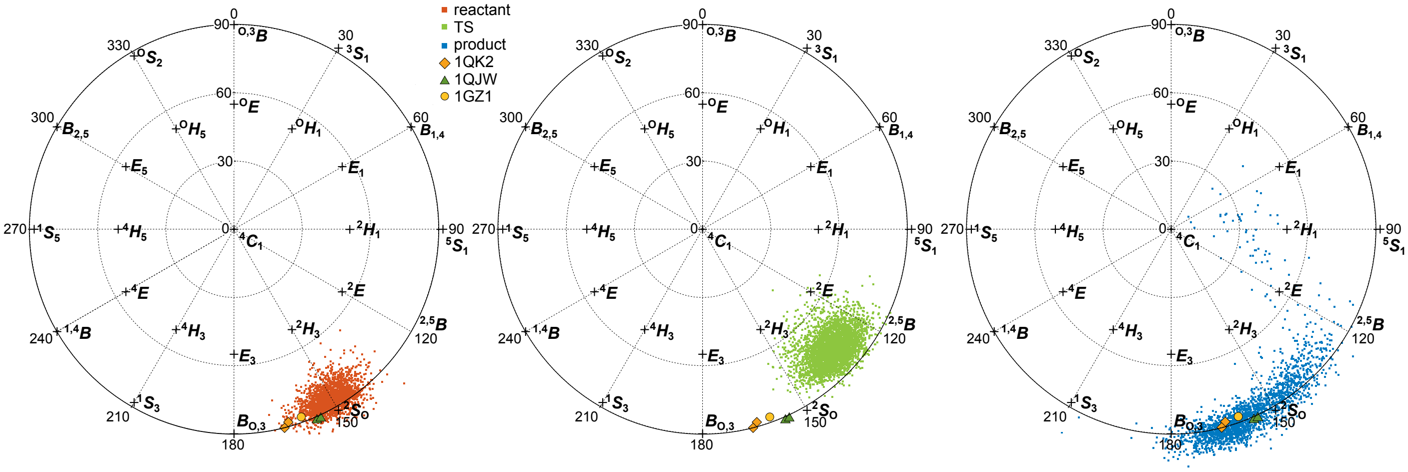

Fig. S5 As in Fig. 7 of the main text but separated into panels for clarity, a top-down view of the northern-hemisphere of the Cremer-Pople sphere[46] designating the -1 glucopyranoside puckering coordinates for the reactant (red), transition state (TS; green), and product (blue) for the elementary reaction shown in Fig. 4 in the main text. Markers designate the puckering coordinates for the -1 glucopyranoside of the substrate mimic methyl 4-S-β-D-cellobiosyl-4-thio-β-D-cellobioside co-crystallized with *Tr*Cel6A wild-type (PDB ID: 1QK2; orange diamond);[47] *Tr*Cel6A Y169F mutant (PDB ID: 1QJW; dark green triangle);[47] and *Humicola insolens* Cel6A D416A mutant (PDB ID: 1GZ1; yellow circle).[48] The two markers for PDB IDs 1QK2 and 1QJW represent the two chains present in the deposited structures. The blue line shows an approximate, smoothed ring-puckering path sampled during fluctuations of the product ring conformation.

Likelihood Maximization

The best-scoring OPs are list in Table S2 and indicate the importance of the distances nucleophilic and bridge water molecules. Interestingly, eight combinations of OPs for the three-parameter RC shared the same likelihood score. Note that these sets of OPs are not independent, as OPs 12 and 13 are combinations of OPs 4, 5, 6, and 7.

Table S2 Best scoring one-, two-, and three-parameter RCs from Likelihood maximization. The Bayesian information criterion is 5.2 for these results.

OPs in RC	likelihood score
12	−11612
4	−12175
6 and 12	−10858
12 and 14	−10930
4, 12, and 13	−10740
4, 6, and 12	−10740
5, 12, and 13	−10740
5, 6, and 12	−10740
4, 5, and 13	−10740
6, 12, and 13	−10740
4, 5, and 6	−10740
4, 6, and 13	−10740

The RC values are calculated as linear combinations of the normalized parameter (q_k) as $RC(\mathbf{q}) = \alpha_0 + \sum_{k=1}^{M} \alpha_k q_k$. In the case of the RC consisting of OP6 and OP12, the associated parameter values are: $\alpha_0 = -6.392$ Å^{-1}, $\alpha_1 = 2.937$ Å^{-1}, and $\alpha_2 = 11.773$ Å^{-1}. Normalization of the OPs was calculated based on the OP6 range of 0.915 to 3.600 Å, and OP12 range of -2.096 to 0.535 Å. The histogram resulting from the committor analysis test is shown in Fig. S6.

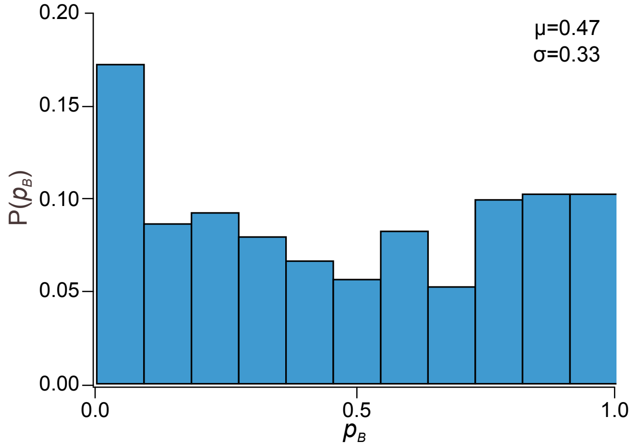

Fig. S6 The p_B histogram for the two-parameter RC (OP6 and OP12) does not display the ideal sharply peaked, binomial distribution centered at p_B=0.5.

The histogram shown in Fig. S6 is used to quantify the accuracy of the reaction coordinate.[49] Optimally, the histogram would be sharply peaked at p_B=0.5, corresponding to a tight distribution around the characteristic isocommittor value for transition states.[50] Sampling errors and the complexity of the reaction coordinate contribute to deviations from ideal results.[16,31] We calculated the transmission coefficient to correct the rate calculated coefficient from this putative reaction coordinate.

D175A Mutant Hydrolysis

As previously described, two simulation systems were used to investigate hydrolysis reactions in the *Tr*Cel6A D175A mutant. The system for which an additional water molecule was included in the QM region (additional compared to the WT QM region) was described in the main text. Here, we present results for the case in which the QM region was the same as for the WT except for the exclusion of the mutated residue 175. As described in the main text for the simulations with an additional water, guesses for TS conformations were generated with biased MD simulations based on sets of distances for OP1–6 harvested from several well-equilibrated (more than 2,000 trajectories completed), accepted WT AS points for use in targeted MD to create TS guesses for the D175A mutant cases. From these guesses, we were able to obtain multiple reactive trajectories for hydrolysis of the glycosidic bond.

Fig. S7 shows the hydrolysis reaction observed for this simulation system: a proton was transferred from D221 to the glycosidic oxygen and the glycosidic bond between the substrate glycosyl units in the −1 and +1 positions was cleaved. The reaction path initially closely followed that in the WT; the glycosidic bond elongated, D221 transferred a proton to the glycosidic oxygen, a water molecule attacked the anomeric carbon at the −1 substrate position, and then transferred a proton to the second water molecule in the QM region. However, the excess proton was not able to transfer beyond the bridge water due to the boundaries of the QM region used in the simulation. Instead, it exchanged between the −1 glucopyranoside ring anomeric hydroxyl group and the bridge water, as shown in the right-hand panel of Fig. S7. As described in the main text, the simulations with an additional water molecule show that further proton transfer is possible if additional potential proton acceptors are included in the QM region.

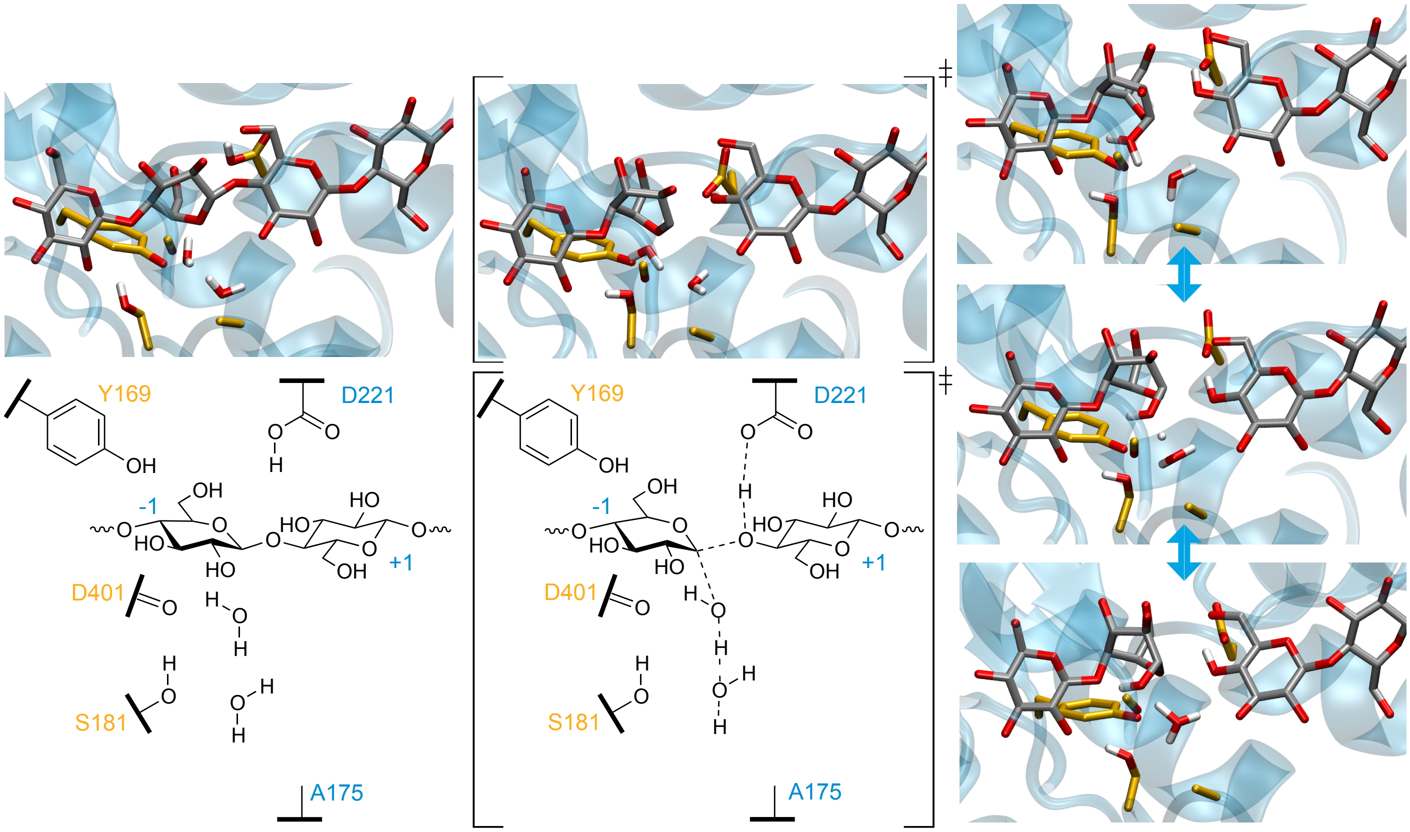

Fig. S7 Modeling the one-step inverting hydrolysis for the *Tr*Cel6A D175A mutant with a QM region as in the WT case, excluding residue 175 and not including any other molecules or residues, yielded reactant (left) and TS (middle) conformations are similar to those for the WT case. However, without a low-energy proton acceptor in the QM region, the proton shuttled between the -1 glucopyranoside ring anomeric hydroxyl group and the "bridge" water in the "product" state (right).

Processivity

Fig. S8 provides a comparison between configurations near the end of the 40 ns US processivity simulations with reference structures built from crystal structures deposited in the PDB. For both the "pre-slide" and "slide" conformations, the reference WT protein structure came from PDB ID: 1QK2[47] with the cellohexaose substrate from PDB ID: 4AVO[51] structure, aligned to the substrate mimic co-crystalized with WT protein in the PDB code 1QK2. As described in the Computational Methods, the "pre-slide" substrate initial conformation was created by removing the monomers in the -2 and -1 substrate binding sites and adding monomers in the $+5$ and $+6$ positions with the same initial geometry as the $+3$ and $+4$ monomers. The simulation and reference crystal structure conformations were aligned in VMD based on all protein backbone atoms. The figure shows alignment of the substrate binding sites as well as expected differences in conformation resulted from the equilibration and simulation of the system, especially for the $+5$ and $+6$ substrate subunits in the "pre-slide" conformation. As noted, the conformation of these monomers was not obtained from the crystal structure and these subunits have greater flexibility as they extend beyond the active site tunnel.

Fig. S9 shows the total interaction energies of individual key protein residues with the cellohexaose substrate as a function of the CV for processivity (the RMSD in Å between the configuration in the simulation and a reference configuration in the "pre-slide" conformation). The magnitude of the energetic interactions for the charged residues shown in panels A and B (note that D221 is protonated and therefore neutral) are primarily due to electrostatic interactions, while the magnitude of the energetic interactions for the aromatic groups in panel C are primarily van der Waals interactions. Fig. 3 in the main text shows the location of the hydrophobic residues in the active site tunnel, whose interactions with the substrate are shown in panel C. Fig. S10 shows the locations of the residues highlighted in Figure S9A and B. Fig. S11 and Fig. S12 show interactions of individual substrate glycosyl groups with residues with the strongest interaction energies.

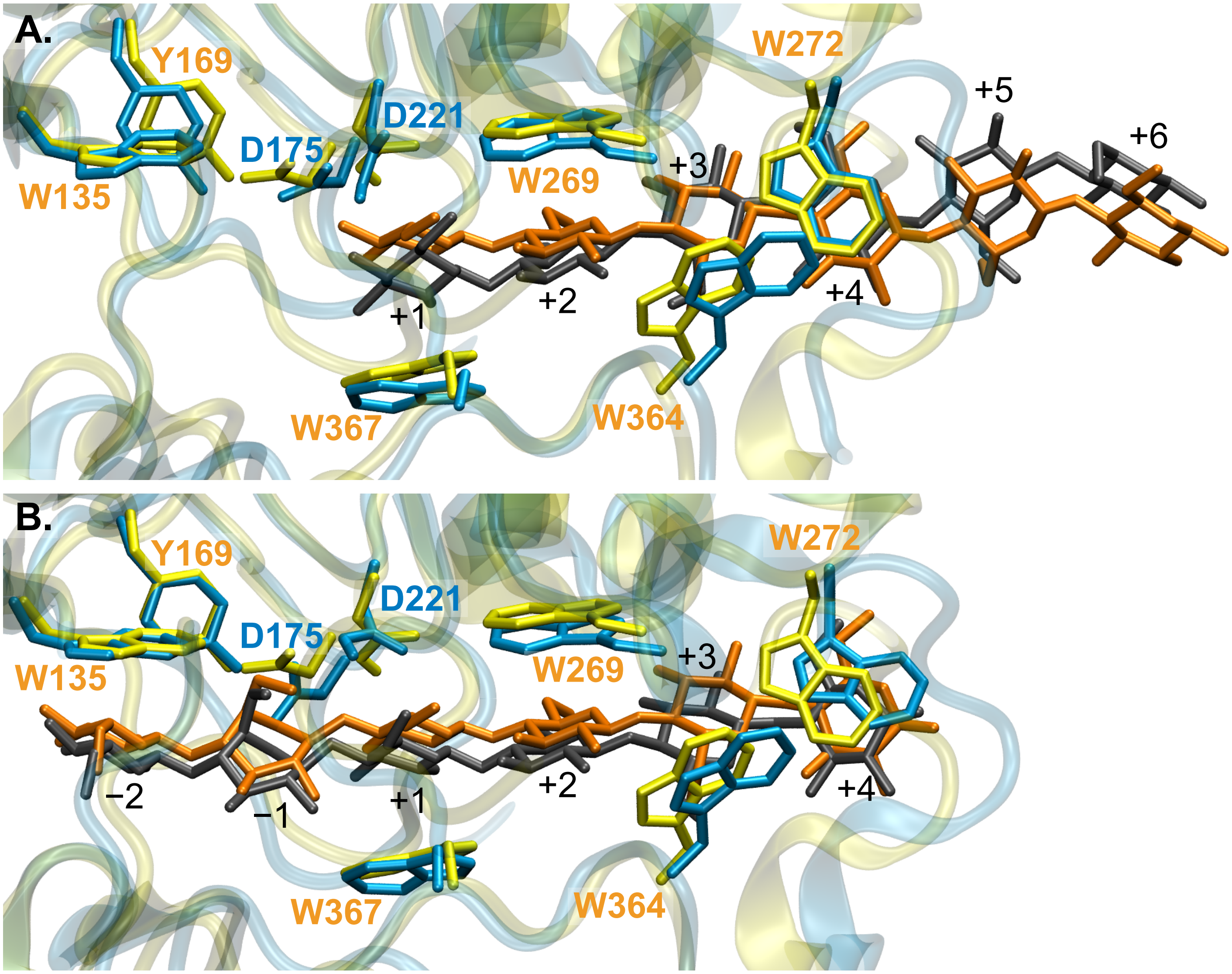

Fig. S8 Conformations from processivity US simulations (blue protein and gray substrate) compared to reference crystal structures (yellow protein and orange substrate). (A) The "pre-slide" conformation was taken from near the end of the US simulations from the window centered at 0.0 Å and has a CV value of 0.2 Å. (B) The "slide" conformation was taken from near the end of the US simulation from the window centered at 10.5 Å and has a the CV value of 10.4 Å, the value of the minimum on the PMF shown in the main text Fig. 10.

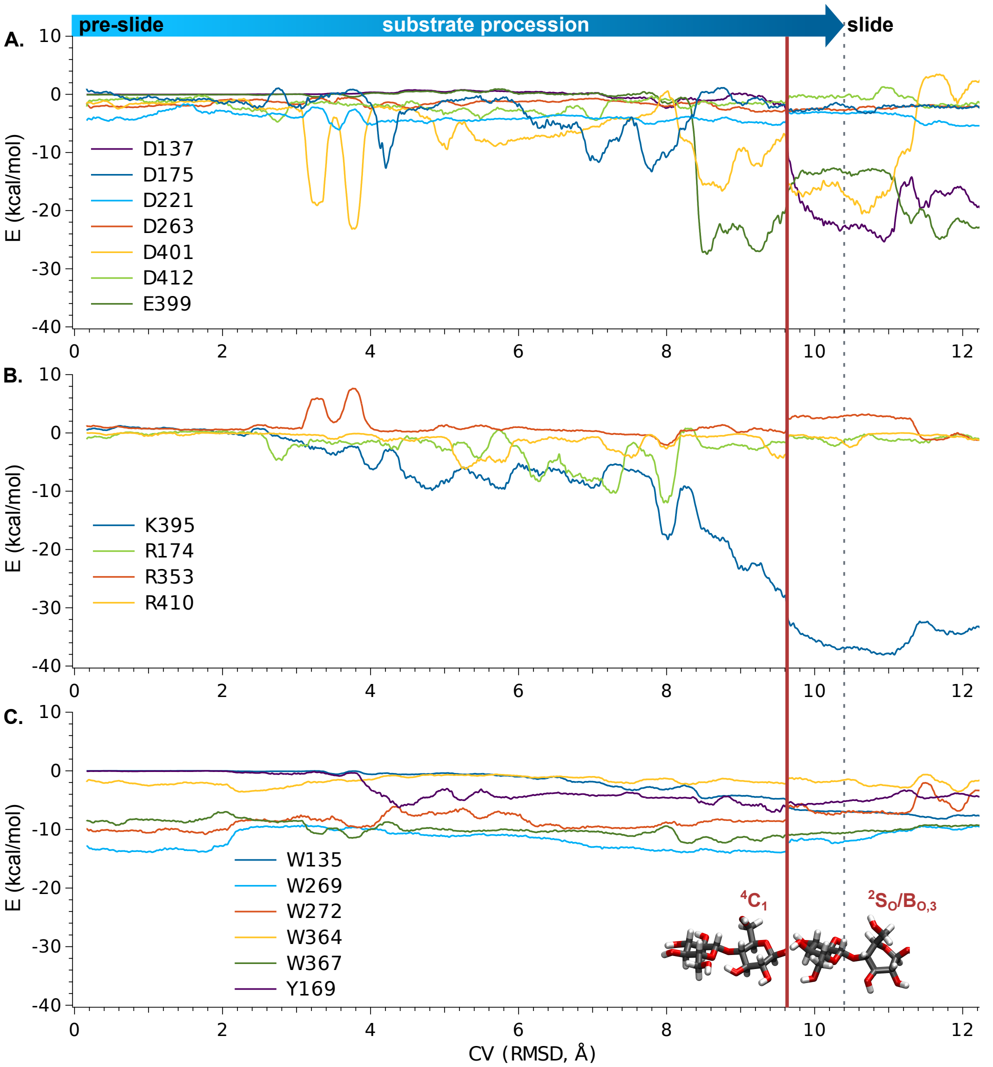

Fig. S9 Total interaction energies between the entire substrate and individual residues labeled in each plot: (A) aspartic and glutamic acids in the active site tunnel are negatively charged, except for D221; (B) positively charged residues in the active site tunnel; and (C) hydrophobic residues in the active site tunnel. The x-axis represents the CV for procession through the active-site containing tunnel: the RMSD of the leading cellobiose ring atom coordinates to a reference conformation in the pre-slide conformation. The dotted gray line indicates the point along the CV corresponding to the slide conformation, at which point the substrate is in position in the active site for hydrolysis. The solid red line designates a puckering dividing line; to the left of the line, the second glycosyl unit is exclusively in the $^4C_1$ conformation and to the right it is only in the $^2S_O/B_{O,3}$ conformation. The data was smoothed as described in the Computational Methods in the main text.

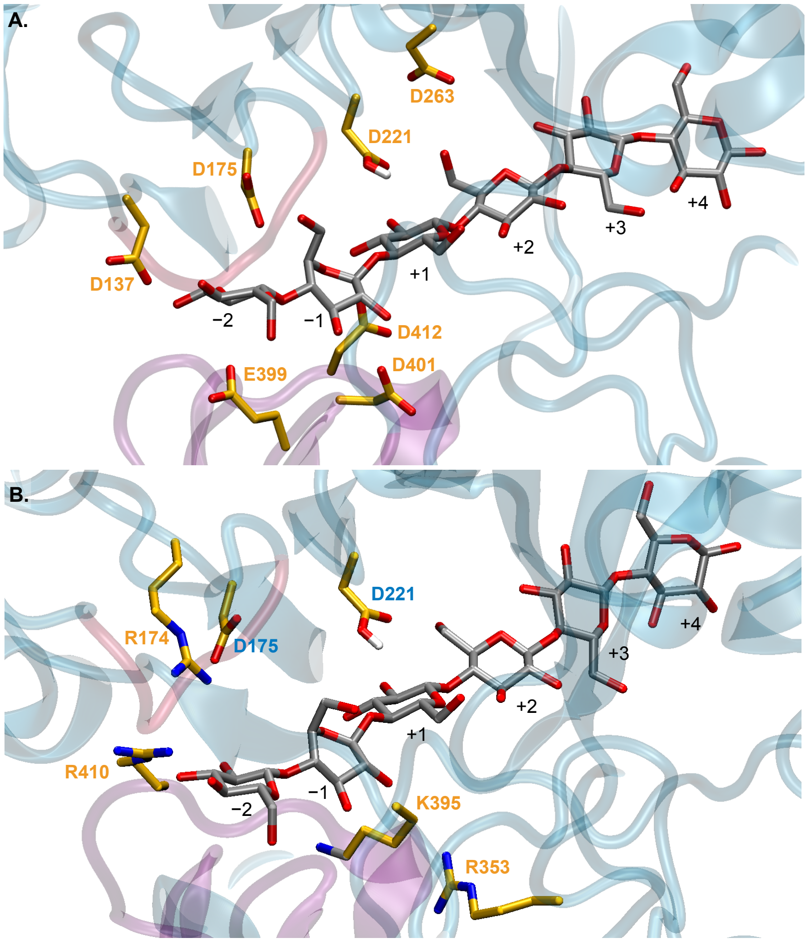

Fig. S10 Cross-sectional review of the *Tr*Cel6A tunnel in the slide modes, with residue side chains noted and labeled which appear in (A) S9 panel A and (B) S9 panel B. The substrate binding sites are labeled in black.

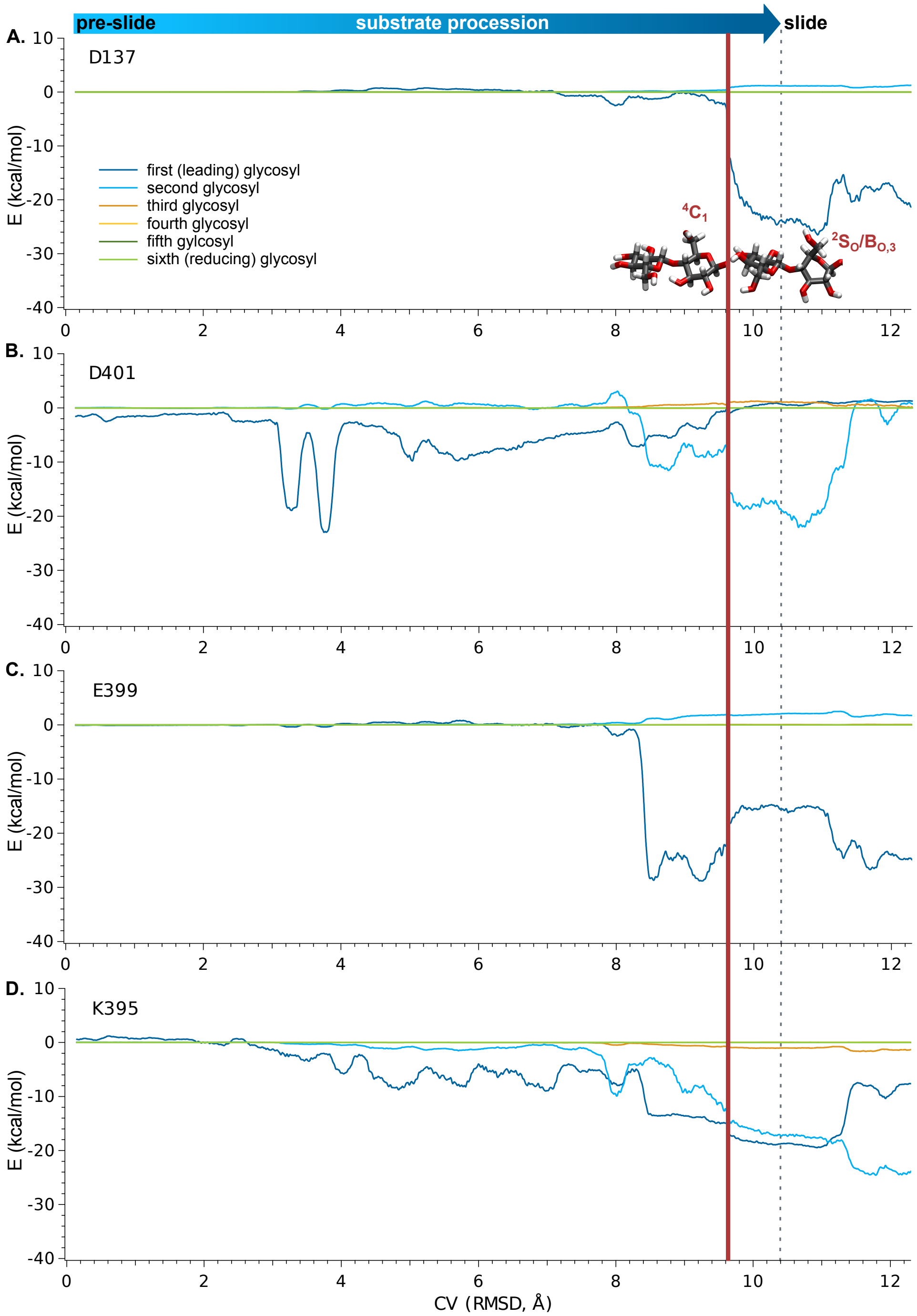

Fig. S11 Total interaction energies between the individual substrate glycosyl units (as labeled) and key charged residues. As in Fig. S9, the x-axis represents the CV for procession through the active-site containing tunnel, the dotted gray line indicates the CV value corresponding to the slide conformation, and the solid red line designates a puckering dividing line. The data was smoothed as described in the Computational Methods in the main text.

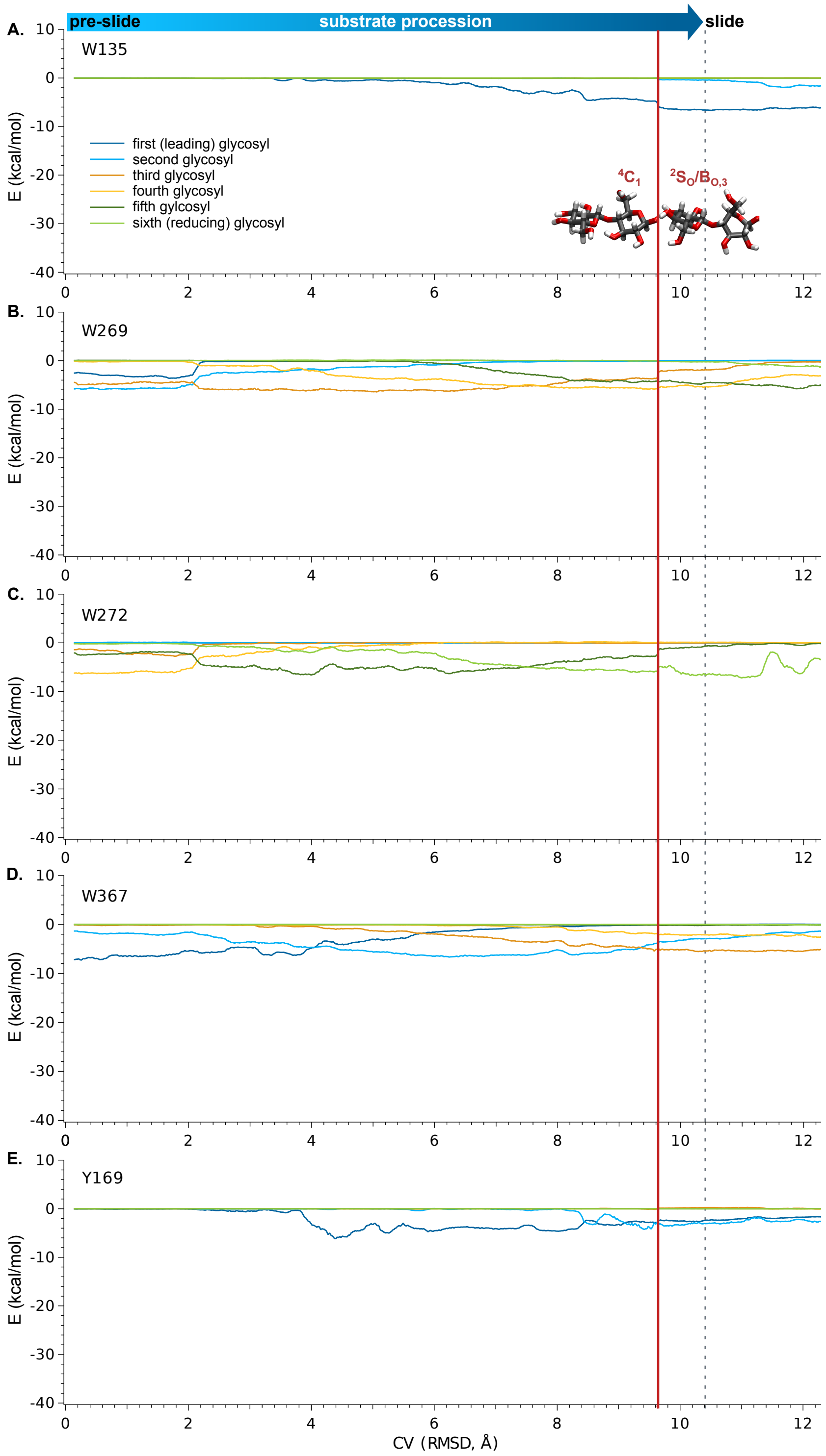

Fig. S12 Total interaction energies between the individual substrate glycosyl units (as labeled) and key hydrophobic residues. As in Fig. S9, the x-axis represents the CV for procession through the active-site containing tunnel, the dotted gray line indicates the CV value corresponding to the slide conformation, and the solid red line designates a puckering dividing line. The data was smoothed as described in the Computational Methods in the main text.

References

1 R. B. Best, X. Zhu, J. Shim, P. E. M. Lopes, J. Mittal, M. Feig and A. D. Mackerell, Jr., *J. Chem. Theory Comput.*, 2012, **8**, 3257–3273.

2 A. D. MacKerell, Jr., M. Feig and C. L. Brooks, III, *J. Am. Chem. Soc.*, 2004, **126**, 698–699.

3 A. D. Mackerell, Jr., D. Bashford, M. Bellott, R. L. Dunbrack, Jr., J. D. Evanseck, M. J. Field, S. Fischer, J. Gao, H. Guo, S. Ha, D. Joseph-McCarthy, L. Kuchnir, K. Kuczera, F. T. K. Lau, C. Mattos, S. Michnick, T. Ngo, D. T. Nguyen, B. Prodhom, W. E. Reiher, III, B. Roux, M. Schlenkrich, J. C. Smith, R. Stote, J. Straub, M. Watanabe, J. Wiórkiewicz-Kuczera, D. Yin and M. Karplus, *J. Phys. Chem. B*, 1998, **102**, 3586–3616.

4 O. Guvench, S. N. Greene, G. Kamath, J. W. Brady, R. M. Venable, R. W. Pastor and A. D. Mackerell, Jr., *J. Comput. Chem.*, 2008, **29**, 2543–2564.

5 O. Guvench, E. Hatcher, R. M. Venable, R. W. Pastor and A. D. Mackerell, Jr., *J. Chem. Theory Comput.*, 2009, **5**, 2353–2370.

6 D. Beglov and B. Roux, *J. Chem. Phys.*, 1994, **100**, 9050–9063.

7 W. L. Jorgensen, J. Chandrasekhar, J. D. Madura, R. W. Impey and M. L. Klein, *J. Chem. Phys.*, 1983, **79**, 926–935.

8 B. R. Brooks, C. L. Brooks, III, A. D. Mackerell, Jr., L. Nilsson, R. J. Petrella, B. Roux, Y. Won, G. Archontis, C. Bartels, S. Boresch, A. Caflisch, L. Caves, Q. Cui, A. R. Dinner, M. Feig, S. Fischer, J. Gao, M. Hodoscek, W. Im, K. Kuczera, T. Lazaridis, J. Ma, V. Ovchinnikov, E. Paci, R. W. Pastor, C. B. Post, J. Z. Pu, M. Schaefer, B. Tidor, R. M. Venable, H. L. Woodcock, X. Wu, W. Yang, D. M. York and M. Karplus, *J. Comput. Chem.*, 2009, **30**, 1545–1614.

9 U. Essmann, L. Perera, M. L. Berkowitz, T. Darden, H. Lee and L. G. Pedersen, *J. Chem. Phys.*, 1995, **103**, 8577–8593.

10 D. A. Case, T. E. Cheatham, III, T. Darden, H. Gohlke, R. Luo, K. M. Merz, Jr., A. Onufriev, C. Simmerling, B. Wang and R. J. Woods, *J. Comput. Chem.*, 2005, **26**, 1668–1688.

11 D. A. Case, T. A. Darden, T. E. Cheatham, III, C. L. Simmerling, J. Wang, R. E. Duke, R. Luo, R. C. Walker, W. Zhang, K. M. Merz, B. Roberts, S. Hayik, A. Roitberg, G. Seabra, J. Swails, A. Götz, I. Kolossváry, K. F. Wong, F. Paesani, J. Vanicek, R. M. Wolf, J. Liu, X. Wu, S. R. Brozell, T. Steinbrecher, H. Gohlke, Q. Cai, X. Ye, J. Wang, M.-J. Hsieh, G. Cui, D. R. Roe, D. H. Mathews, M. G. Seetin, R. Salomon-Ferrer, C. Sagui, V. Babin, T. Luchko, S. Gusarov, A. Kovalenko and P. A. Kollman, *AMBER 12*, 2012.

12 T. A. Andrea, W. C. Swope and H. C. Andersen, *J. Chem. Phys.*, 1983, **79**, 4576–4584.

13 M. F. Crowley, M. J. Williamson and R. C. Walker, *Int. J. Quantum Chem.*, 2009, **109**, 3767–3772.

14 P. G. Bolhuis, D. Chandler, C. Dellago and P. L. Geissler, *Annu. Rev. Phys. Chem.*, 2002, **53**, 291–318.

15 B. Peters, G. T. Beckham and B. L. Trout, *J. Chem. Phys.*, 2007, **127**, 034109.

16 G. T. Beckham and B. Peters, *Computational Modeling in Lignocellulosic Biofuel Production*, American Chemical Society, 2010, ch. 13, pp. 299–332.

17 G. T. Beckham, J. Ståhlberg, B. C. Knott, M. E. Himmel, M. F. Crowley, M. Sandgren, M. Sørlie and C. M. Payne, *Curr. Opin. Biotechnol.*, 2014, **27**, 96–106.

18 C. M. Payne, B. C. Knott, H. B. Mayes, H. Hansson, M. E. Himmel, M. Sandgren, J. Ståhlberg and G. T. Beckham, *Chem. Rev.*, 2015, **115**, 1308–1448.

19 M. Elstner, D. Porezag, G. Jungnickel, J. Elsner, M. Haugk, T. Frauenheim, S. Suhai and G. Seifert, *Phys. Rev. B*, 1998, **58**, 7260–7268.

20 G. d. M. Seabra, R. C. Walker, M. Elstner, D. A. Case and A. E. Roitberg, *J. Phys. Chem. A*, 2007, **111**, 5655–5664.

21 P. Goyal, M. Elstner and Q. Cui, *J. Phys. Chem. B*, 2011, **115**, 6790–6805.

22 M. Elstner, *Theor. Chem. Acc.*, 2005, **116**, 316–325.

23 M. Elstner, T. Frauenheim and S. Suhai, *J. Mol. Struct. THEOCHEM*, 2003, **632**, 29–41.

24 T. Krüger, M. Elstner, P. Schiffels and T. Frauenheim, *J. Chem. Phys.*, 2005, **122**, 114110.

25 M. Hoffmann, M. Wanko, P. Strodel, P. H. König, T. Frauenheim, K. Schulten, W. Thiel, E. Tajkhorshid and M. Elstner, *J. Am. Chem. Soc.*, 2006, **128**, 10808–10818.

26 P. H. König, N. Ghosh, M. Hoffmann, M. Elstner, E. Tajkhorshid, T. Frauenheim and Q. Cui, *J. Phys. Chem. A*, 2006, **110**, 548–563.

27 C. M. Maupin, B. Aradi and G. A. Voth, *J. Phys. Chem. B*, 2010, **114**, 6922–6931.

28 R. Liang, J. M. J. Swanson and G. A. Voth, *J. Chem. Theory Comput.*, 2014, **10**, 451–462.

29 T. H. Choi, R. Liang, C. M. Maupin and G. A. Voth, *J. Phys. Chem. B*, 2013, **117**, 5165–5179.

30 P. Goyal, H.-J. Qian, S. Irle, X. Lu, D. Roston, T. Mori, M. Elstner and Q. Cui, *J. Phys. Chem. B*, 2015, **188**, 11007–11027.

31 B. C. Knott, M. Haddad Momeni, M. F. Crowley, L. F. Mackenzie, A. W. Götz, M. Sandgren, S. G. Withers, J. Ståhlberg and G. T. Beckham, *J. Am. Chem. Soc.*, 2014, **136**, 321–329.

32 K. Igarashi, T. Uchihashi, A. Koivula, M. Wada, S. Kimura, T. Okamoto, M. Penttilä, T. Ando and M. Samejima, *Science*, 2011, **333**, 1279–1282.

33 B. Peters and B. L. Trout, *J. Chem. Phys.*, 2006, **125**, 054108.

34 G. M. Torrie and J. P. Valleau, *J. Comput. Phys.*, 1977, **23**, 187–199.

35 A. Laio and M. Parrinello, *Proc. Natl. Acad. Sci. U.S.A.*, 2002, **99**, 12562–12566.
36 B. Peters, *J. Chem. Theory Comput.*, 2010, **6**, 1447–1454.
37 R. Radhakrishnan and T. Schlick, *J. Chem. Phys.*, 2004, **121**, 2436–2444.
38 R. Lynden-Bell, J. Van Duijneveldt and D. Frenkel, *Mol. Phys.*, 1993, **80**, 801–804.
39 J. C. Phillips, R. Braun, W. Wang, J. Gumbart, E. Tajkhorshid, E. Villa, C. Chipot, R. D. Skeel, L. Kalé and K. Schulten, *J. Comput. Chem.*, 2005, **26**, 1781–1802.
40 J.-P. Ryckaert, G. Ciccotti and H. J. C. Berendsen, *J. Comput. Phys.*, 1977, **23**, 327–341.
41 B. Roux, *Comput. Phys. Commun.*, 1995, **91**, 275–282.
42 A. Grossfield, *WHAM: the weighted histogram analysis method, version 2.0.6*, 2012.
43 W. Humphrey, A. Dalke and K. Schulten, *J. Mol. Graphics*, 1996, **14**, 33–38.
44 B. C. Knott, M. F. Crowley, M. E. Himmel, J. Ståhlberg and G. T. Beckham, *J. Am. Chem. Soc.*, 2014, **136**, 8810–8819.
45 L. Bu, M. F. Crowley, M. E. Himmel and G. T. Beckham, *J. Biol. Chem.*, 2013, **288**, 12175–12186.
46 D. Cremer and J. A. Pople, *J. Am. Chem. Soc.*, 1975, **97**, 1354–1358.
47 J.-y. Zou, G. J. Kleywegt, J. Ståhlberg, H. Driguez, W. Nerinckx, M. Claeyssens, A. Koivula, T. T. Teeri and T. A. Jones, *Structure*, 1999, **7**, 1035–1045.
48 A. Varrot, T. P. Frandsen, H. Driguez and G. J. Davies, *Acta Crystallogr., Sect. D: Biol. Crystallogr.*, 2002, **58**, 2201–2204.
49 B. Peters, *J. Chem. Phys.*, 2006, **125**, 241101.
50 P. L. Geissler, C. Dellago and D. Chandler, *J. Phys. Chem. B*, 1999, **103**, 3706–3710.
51 M. Wu, L. Bu, T. V. Vuong, D. B. Wilson, M. F. Crowley, M. Sandgren, J. Ståhlberg, G. T. Beckham and H. Hansson, *J. Biol. Chem.*, 2013, **288**, 33107–33117.
